# Ecosystem Multifunctionality Regulated by Soil Microbial Activity and Indicator Taxa Versus Biodiversity for Industrial Solar Facilities on the Qinghai–Tibet Plateau

**DOI:** 10.3390/microorganisms13071464

**Published:** 2025-06-24

**Authors:** Yu Liu, Chengxiang Ding, Tiemei Wang, Derong Su, Zhuoqing Li, Chaoyang Feng, Zhanjun Quan

**Affiliations:** 1 State Key Laboratory of Environmental Criteria and Risk Assessment, Beijing 100012, China; ly159632874@163.com; 2 Institute of Ecology, Chinese Research Academy of Environmental Sciences, Beijing 100012, China; 3 Academy of Animal Science and Veterinary Medicine, Qinghai University, Xining 810016, China; 4 School of Grassland, Beijing Forestry University, Beijing 100083, China

**Keywords:** arid region, ecosystem multifunctionality, microbial activity, solar facility, soil microbiome

## Abstract

The drive towards carbon neutrality has prompted the worldwide expansion of utility-scale solar facilities. Previous studies have reported the positive effects of solar facilities’ installation on pasture productivity and biodiversity in arid regions. However, our understanding of how solar facilities influence a wide range of ecosystem functions simultaneously, and the relative contributions of soil microbial attributes, remains incomplete. To address this gap, we assessed the changes in ecosystem multifunctionality following solar facility installation in an alpine desert grassland in the Qinghai–Tibet plateau by measuring twenty-three ecosystem function indicators comprising primary production, the soil nutrient pool, carbon cycling, nitrogen cycling, phosphorus cycling and oxidation–reduction. Furthermore, we estimated the soil microbial diversity, microbial indicator taxa and microbial activity to identify the primary driving factors. The results showed that solar facilities had positive effects on ecosystem multifunctionality; the positive effect size was higher in the initial installation period (31.4%) than in the constant running period (3.5%). The enhancements in ecosystem multifunctionality were mainly due to enhanced nutrient cycling induced by the increased abundance of fungal indicator taxa and microbial activity. Moreover, the structural equation model revealed distinct regulatory paths between the two periods and a transition in the primary driving factors of ecosystem multifunctionality from microbial indicator taxa to microbial activity. In conclusion, our study demonstrates the positive influence of solar facilities on multiple ecosystem functions, emphasizing the critical role of soil microbial mechanisms in regulating ecosystem multifunctionality. These findings provide valuable insights into soil biota-driven processes that could inform strategies aimed at enhancing soil health and ecosystem functionality in arid grasslands under human-managed systems.

## 1. Introduction

The need for carbon neutrality has led to the rapid, global expansion of renewable energy, with photovoltaic solar energy production increasing more than fivefold in the past decade [1], and it is predicted to increase tenfold according to the International Energy Agency [2]. While solar power offers a low-carbon energy source, the construction and operation of large-scale photovoltaic solar facilities (SFs) can significantly alter local ecosystems. Land use changes associated with SFs, including vegetation removal, soil compaction and shading from panels, can disrupt microclimates, soil processes and biotic communities [3]. Such changes could have dramatic implications for microclimates, soil nutrients, vegetation and soil microbiota [4] and further ecosystem functions. For instance, habitats under photovoltaic panels have shown benefits for partial ecosystem functions, such as greater nutrient cycles and lower soil CO_2_ effluxes [5]. Negative impacts have also been reported, such as eliciting biodiversity loss [6], reducing primary production [7] and decreasing soil aggregate stability [5]. These conflicting findings may stem from spatial variability, but, more importantly, most existing studies have focused on isolated ecosystem functions. Consequently, a comprehensive evaluation integrating multiple ecosystem functions, termed ecosystem multifunctionality (EMF), is needed to fully understand the ecological consequences of SFs [8].

EMF integrates multiple ecosystem processes, such as productivity, nutrient cycling and soil stability, into a single framework, and it has become a widely used indicator to assess the ecological impacts of anthropogenic activities [9]. Despite the careful interpretation of the ecological meaning of multifunctionality metrics, this approach can help to quantify the direction and/or degree of changes in EMF. Previous studies have described a prevailing argument that taxonomic diversity is positively correlated with EMF [10], and the loss of taxa in a community could lead to a decline in EMF [11]. However, the concept of functional redundancy challenges this view, suggesting that the loss of species does not necessarily lead to functional declines [12]. Owing to the shared functions among different groups of species, a decrease in species diversity does not necessarily lead to a corresponding decline in ecosystem function [13]. An increasing number of studies have uncovered the disproportionate significance of specific microbial groups in ecosystem processes, such as rare taxa [14], keystone taxa [15] and indicator taxa [16]. These findings highlight the need to move beyond diversity indices and consider microbial traits and metabolic activity as drivers of EMF. Nonetheless, most studies rely on the relative abundance to evaluate microbial communities, which fails to capture shifts in community-level biological activity under disturbed conditions [17]. A growing body of research has found positive effects between microbial metabolic activity and ecosystem functions, such as soil carbon storage and organic matter decomposition [18,19]. These reports indicate that analyzing microbial profiles based on the whole community provides a more realistic perspective than the relative abundance approach. This approach can also offer complementary information to better understand how soil microorganisms regulate ecosystem functions.

Importantly, the ecological impacts of SFs are likely to differ between the installation phase, characterized by intense soil disturbance, and the operation phase, where ongoing microclimatic alterations predominate [20]. However, few studies have systematically compared how the regulatory mechanisms of EMF shift over these stages. Understanding such temporal dynamics is crucial in assessing the long-term ecological sustainability of SFs. To fill this gap, soils from three habitats with different aging periods were collected. Twenty-three ecosystem functions—including primary productivity; soil nutrient content (total and available), soil carbon, nitrogen and phosphorus cycling; and oxidation–reduction functions—were measured to represent EMF. We analyzed the relationship between EMF and the potential driving factors, including the taxonomic, phylogenetic and functional diversity indices of plants, prokaryotes and fungi; specific types of microbial taxa; microbial biomass; metabolic activity; and environmental factors. Then, we quantified their relative contributions and determined the dominant driving factors for EMF based on multiple statistical models and machine learning measures. Lastly, we investigated the regulatory mechanisms of EMF during different periods.

The primary goal of this study was to improve our understanding of how utility-scale solar facilities influence EMF in arid alpine grasslands, with a particular focus on disentangling the relative contributions of microbial, plant and environmental factors to changes in EMF. To achieve this, we addressed the following specific objectives: (1) quantify the effects of SF installation and operation on multiple ecosystem functions and overall multifunctionality; (2) identify the primary biotic and abiotic drivers shaping EMF changes associated with SF development; (3) elucidate the ecological mechanisms underlying shifts in EMF across different stages of SF installation and operation.

## 2. Materials and Methods

### 2.1. Site Description, Experimental Design and Soil Sampling

**Figure 1:**
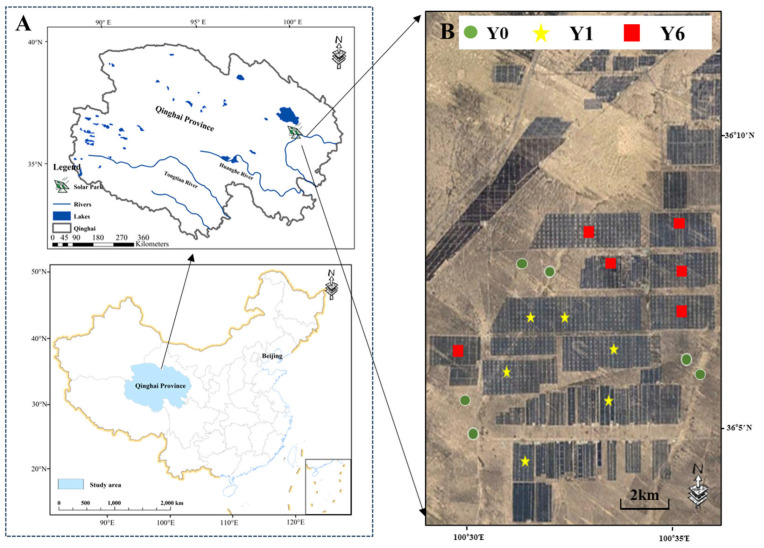
(**A**) Location of solar facility; (**B**) satellite image of sample sites.

The study was conducted at the Longyangxia Dam Solar Facility (Figure 1A). Established in 2013, Longyangxia Dam Solar Park, with a capacity of 850 MW, is situated in the southeastern region of the Qinghai–Tibet Plateau (36°9′47″ N,100°35′14″ E). The ecosystem type is an alpine desert grassland characterized by an average annual temperature ranging from 4.8 to 6.0 °C, average annual precipitation of 278–523 mm and annual evaporation of 1716.7 mm. This climatic region is classified as semi-arid. The predominant soil texture is primarily sandy loam. Within this environment, the dominant plant species include *Leymus chinensis*, *Achnatherum splendens* and *Artemisia frigida*. Before being transformed into a solar facility, the field site underwent destructive measures, including soil leveling and the removal of plants.

To avoid the impacts of the environmental heterogeneity of native habitats, we firstly measured the pre-installation environmental conditions based on eleven indicators using remote sensing; the Supplementary Materials provide detailed information on the data sources and analysis methods. Then, eighteen plots without significant environmental differences were selected to evaluate the impacts of solar facilities on EMF (Figure 1B and Figure S1). Among them, six plots were assessed one year after SF installation, six plots were assessed six years after SF installation and six plots were in natural areas without SF installation.

In mid-August in 2019, we established a quadrat (50 cm × 50 cm) in each plot. The height and specific leaf area of each plant species were measured. The number of each plant species in a quadrat was recorded to estimate the species diversity and community structure. The plant aboveground part was clipped to measure the aboveground biomass; the belowground parts were collected to measure the belowground biomass.

Then, five soil cores were collected from each quadrat at a depth of 0–10 cm. To reduce variability caused by spatial heterogeneity, soil cores from the same quadrat were combined into a composite sample. Each composite sample was then divided into subsamples for separate microbial and chemical analyses. For microbial analysis, samples were placed in sterile bags to prevent contamination and stored at −20 °C for DNA extraction. For chemical analysis, samples were placed in plastic bags and stored at 4 °C. Various parameters, including the microclimate, vegetation, soil physicochemical properties and soil microorganism metrics, were measured at each site.

### 2.2. Environmental Characteristic Measurements

The soil pH was determined by mixing fresh soil with water in a ratio of 1:5, and measurements were taken using a Sartorius pH meter (PB–10, Sartorius Corporate Administration GmbH, Göttingen, Germany). The soil water content (SWC) was assessed by subjecting the soil to drying at 105 °C for 24 h. The soil temperature (ST) at a depth of 10 cm was recorded using a Hydra Probe sensor (Campbell Scientific, Inc., Logan, UT, USA). Evaporation was measured with the evaporating dish method [21]. The soil texture was determined using sedimentation with the hydrometer method [22].

### 2.3. DNA Extraction and Sequence Processing

Soil DNA was extracted from 0.5 g frozen soil samples using the FastDNA SPIN Kit for Soil (MP Biomedicals, Santa Ana, CA, USA), following the provided instruction manual. Quantification of the extracted DNA was performed using a NanoDrop 2000 Spectrophotometer (Bio-Rad Laboratories Inc., Hercules, CA, USA). PCR was carried out to amplify the prokaryotic 16s rRNA gene and fungal ITS region using the primer set 515F and 806R. Approximately 250 bp paired-end reads were generated on the Illumina MiSeq platform. QIIME2 (version 2018.6) [23] was used to analyze the raw sequencing data. The DADA2 plugin [24] in QIIME2 was employed to filter out low-quality reads and chimeras, generating amplicon sequence variants (ASVs). The taxonomic classification of ASVs was achieved using the QIIME2 naive Bayes classifier trained on the SILVA rRNA database (v132) and the UNITE database for prokaryotes and fungi, respectively. To enhance the robustness of the analysis, only ASVs present in at least 20% of the total samples and containing a minimum of 4 counts in each sample were retained. All subsequent data analysis was conducted based on the filtered dataset.

### 2.4. Biodiversity Measurement

We calculated taxonomic, phylogenetic and functional diversity metrics to represent multiple scales and aspects of biodiversity in each site. All calculations were conducted in R 4.1.2. The taxonomic α-diversity of plants, prokaryotes and fungi was represented by the richness index for plant species and ASVs for prokaryotes and fungi. For fungi and prokaryotes, we constructed phylogenetic trees using the aligned and preprocessed sequences. For the estimation of plant phylogenetic diversity, we initially standardized the scientific names of these plant species using the U.Taxonstand package. Following this, we constructed plant phylogenetic trees using the V.PhyloMaker2 package. Faith’s phylogenetic diversity was subsequently computed based on the generated phylogenetic trees. Soil fungal functions were obtained from the predicted results of FUNguild; only highly probable and probable guilds were used for these analyses. The richness of fungal guilds was used as a proxy for fungal function diversity. The prokaryotic functions were obtained from the predicted results of PICRUST2 [25]. The richness index of enzyme metabolic pathways on the basis of Kyoto Encyclopedia of Genes and Genomes orthologs (KOs) represented the prokaryotic functional diversity. We measured two common functional traits, namely the plant height and specific leaf area (SLA), of each plant species in our study region and then calculated the plant functional diversity based the Gower distance of functional traits using the FD package [26]. We used functional dispersion as a proxy for plant functional traits given its stability to outliers and the strong correlations (>0.7) with most other functional diversity indices (Figure S2).

### 2.5. Microbial Indicator Taxa and Microbial Activity Measurement

We filtered four distinct categories of taxa within both prokaryotes and fungi, which encompassed indicator, keystone, abundant and rare taxa. This approach allowed us to assess the impact of each specific category of taxa on ecosystem functions. Indicator taxa reflect the associations between a species and a group of sites and were filtered using the “indicspecies” package [27]. Network keystone taxa are characterized by their within-module connectivity and among-module connectivity in a microbial co-occurrence network; when a node had Zi  >  2.5 or/and Pi  >  0.62, it was named a network keystone taxon [28]. Abundant and rare taxa were characterized by their relative abundance; ASVs with relative abundances less than 0.01% of the total sequences were regarded as rare taxa, and those with relative abundances greater than 0.1% were determined as abundant taxa [29,30].

We used the gene copy number and microbial biomass carbon (MBC) as a proxy for microbial biomass. The gene copy numbers of total prokaryotes (using the 16S rRNA gene) and fungi (using the internal transcribed spacer region) were quantified using a CFX-96 thermocycler (Bio-Rad), as described previously [31]. MBC was determined using the chloroform fumigation–extraction method [32]. We used the microbial metabolic quotient and the ratio of microbial biomass and soil organic carbon (C_mic_/C_org_) as proxies for microbial metabolic efficiency. The microbial metabolic quotient was quantified using the ratio of carbon mineralization and microbial biomass carbon [33]. These indices provide a comprehensive representation of microbial activity, capturing both the structural (microbial biomass) and functional (metabolic efficiency) aspects of microbial communities that underpin their contributions to ecosystem processes.

### 2.6. Ecosystem Functions and Multifunctionality

In this study, twenty-three environmental and biological characteristics are included, according to the ecosystem functions framework [34], to represent ecosystem functions regulated by plant and soil microorganisms. All these functions are classified into six functional categories: primary production (PP), the soil nutrient pool (SNP), soil carbon cycling (C cycling), soil nitrogen cycling (N cycling), soil phosphorus cycling (P cycling) and oxidation–reduction (redox). The detailed rationale and measurement methods for each variable are provided in the Supplementary Materials. To obtain a quantitative EMF index, we used two independent EMF metrics: (1) the average multifunctionality index (avgFunc) [35] and (2) the multithreshold multifunctionality index [36]. To estimate the average EMF index, we initially standardized each ecosystem function, scaling them between 0 and 1. Subsequently, we computed their mean values. Given that multiple thresholds could provide a better understanding of how driving factors affect ecosystem functions, and to account for potential trade-offs between the functions, we measured multifunctionality with thresholds between 20% and 80% (every 20%).

To distinguish between the ecological impacts of different phases of SF development, we conducted separate assessments to evaluate the annual effect sizes of SFs on ecosystem functions and EMF indices during two distinct periods: the installation period and the running period. The installation period refers to the ecological changes observed between the pre-installation baseline (Y0) and the first year after photovoltaic panel installation (Y1), primarily capturing the effects of construction-related disturbances. The running period represents the ecological changes between the first year (Y1) and the sixth year after installation (Y6), reflecting the long-term impacts associated with microclimatic modifications induced by the sustained presence of solar panels. The effect size of SFs was quantified using the following formula:

(1) $$Effect=\frac{\left(Y_{i}-Y_{j}\right)}{\left(i-j\right)\times Y_{j}}$$

To estimate installation effects, *i* = 1 and *j* = 0. To estimate running effects, *i* = 6 and *j* = 1.

### 2.7. Statistical Analyses

To compare the significant differences in environmental, diversity and ecosystem function and EMF indices between Y0, Y1 and Y6, we conducted a non-parametric test (Kruskal–Wallis H test) using the “stat” package, given that several factors did not follow a normal distribution. To evaluate the relationships between diversity and ecosystem functions, we first carried out ordinary least squares linear regressions between soil taxonomic, phylogenetic and functional alpha diversity and single ecosystem functions and multifunctionality indices. Then, the Mantel test based on “Spearman” with 999 permutations was conducted with the “vegan” package to estimate the correlations between beta diversity, ecosystem functions and multifunctionality indices. Principal component analysis (PCA) was used to reduce the dimensions of the alpha diversity index and indicators for subsequent analysis.

We divided all factors into four categories, namely microbial activity, biological diversity, environmental factors and microbial indicator taxa, to disentangle the critical factors driving EMF (Table 1). To evaluate the relative contribution of each group of factors to ecosystem functions and multifunctionality, a variation partitioning analysis was first conducted to quantify the shared and unique contributions of each group of factors. Furthermore, the relative importance of each factor was calculated based on a multiple linear regression model with the “relaimpo” package. Given the strong intercorrelations among the various factors, a partial correlation analysis was used to evaluate the relationships between the ecosystem functions and the various factors with the “ppcor” package. Each single factor was separately set as the controlling factor to explore the relationship between the ecosystem function and other factors. To disentangle the ways in which SFs affect ecosystem multifunctionality, we constructed a piecewise structural equation model to test the direct and indirect causal relationships among each group of factors and multifunctionality for the SF installation period and running period, respectively. The piecewise structural equation model provides a versatile mathematical framework that alleviates certain constraints commonly associated with conventional structural equation models, such as the non-normal distribution of data [37]. The conceptual structural equation model was constructed and tested using the “piecewiseSEM” package.

## 3. Results

### 3.1. Annual Effect Size of Solar Facilities on Ecosystem Multifunctionality

**Figure 2:**
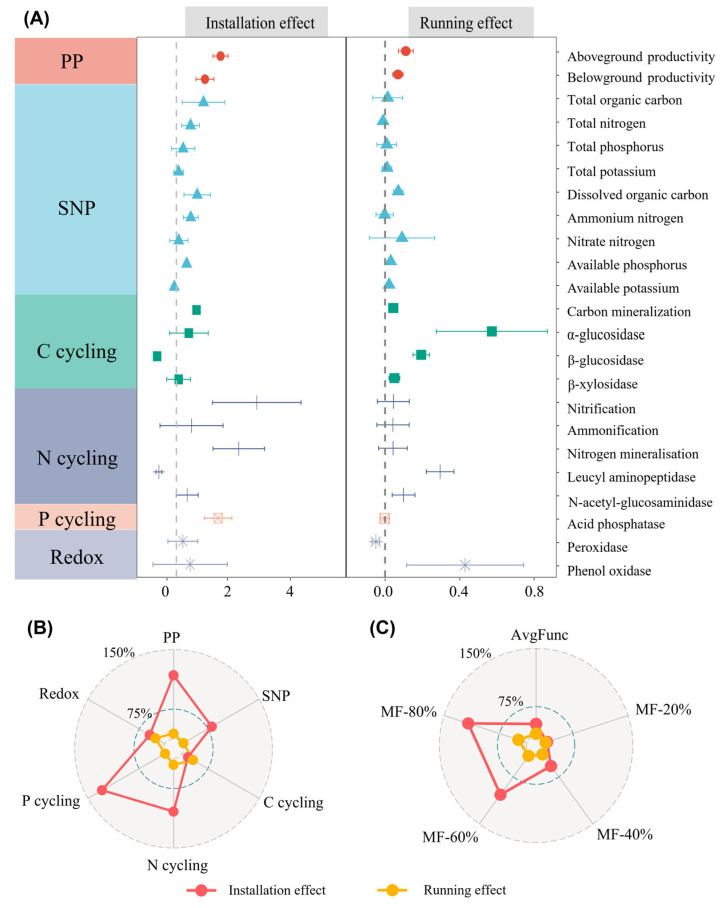
(**A**) The annual effects of solar facilities on each component of ecosystem functions (mean ± 95% confidence intervals); (**B**) The annual effects of solar facilities on six categories of ecosystem functions; (**C**) The annual effects of solar facilities on ecosystem multifunctionality indices. PP indicates primary productivity, SNP indicates soil nutrient pool, C cycling indicates soil carbon cycling, N cycling indicates soil nitrogen cycling, P cycling indicates soil phosphorus cycling and Redox indicates soil oxidation–reduction. AvgFunc indicates average multifunctionality; MF-20%, MF-40%, MF-60% and MF-80% indicate EMF at the 20%, 40%, 60% and 80% threshold, respectively.

Both solar farm installation and solar farm running led to enhancements in diverse ecosystem functional categories and ecosystem multifunctionality (EMF) indices, while the annual effect size was higher in the installation period when compared to that in the running period (Figure 2A–C). Among twenty-three specific functional indicators, solar farm installation exhibited positive and significant effects on aboveground biomass, belowground biomass, total organic carbon, dissolved organic carbon, carbon mineralization, nitrogen mineralization, and acid phosphatase, while it showed significant and negative impacts on leucine aminopeptidase and β-glucosidase (*p* < 0.05) (Figure 2A). Solar farm running resulted in a significant increase in aboveground biomass, belowground biomass, soil hydrolytic enzyme activity related to carbon, and nitrogen cycling, while it exhibited a significant negative effect on peroxidase (*p* < 0.05) (Figure 2A).

In terms of the differences in functional categories between the two phases, solar facilities (SFs) had the most substantial impact on primary productivity at 106%, with the lowest effect on carbon cycling at 11% during the installation period. SFs had the strongest effect on carbon cycling at 21% but exhibited the lowest effect on P cycling, with a slight decrease of −0.1% (Figure 2B) during the running period. Regarding EMF indices, SF installation showed its strongest effect at MF-80% (149.4%), a moderate effect on avgFunc (31.4%), and the weakest at MF-20% (5.4%). Similarly, SF running exhibited the strongest effect at MF-80% (15%), followed by a smaller effect on avgFunc (3.5%) and a minimal effect at MF-20% (0.4%). In addition, the majority of ecosystem functions and EMF metrics exhibited a consistent increase with the time since SF installation, with Y6 displaying a higher value than Y1 and Y0 (Figures S3–S5).

### 3.2. Responses of Diversity to Solar Facilities

**Figure 3:**
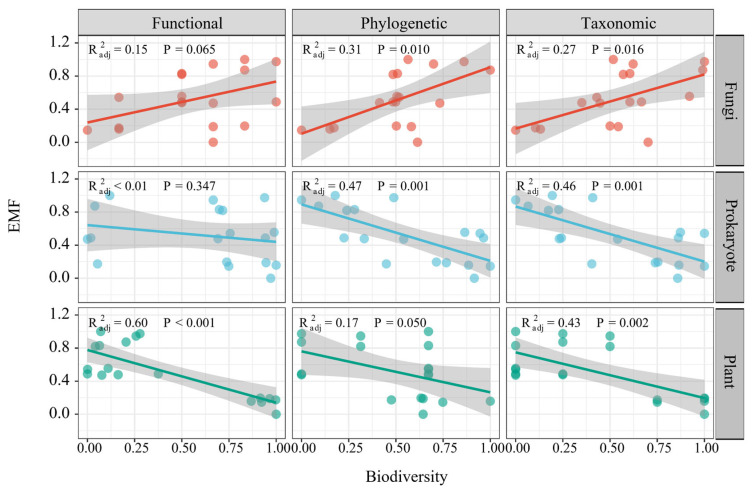
The linear effects of plant, prokaryotic and fungal diversity on ecosystem multifunctionality.

Over the years following SF installation, there were declines in alpha-scale functional, phylogenetic and taxonomic diversity among prokaryotes and plants, while fungi showed an increase in these diversity measures (Figure S6). EMF exhibited positive correlations with the alpha diversity matrices of fungi, encompassing functional, phylogenetic and taxonomic diversity, while it had negative correlations with those of prokaryotes and plants (Figure 3).

### 3.3. Effects of Microbial Taxa on Ecosystem Multifunctionality

We identified 63 indicator taxa, 115 keystone taxa, 114 abundant taxa and 259 rare taxa among fungi. For prokaryotes, we identified 185 indicator taxa, 327 keystone taxa, 299 abundant taxa and 268 rare taxa. With the exception of EMF at the 20% threshold, the mean and 95% confidence interval of the standard linear effects of fungal indicators on all EMF indices were greater than zero, and they were greater than the mean effects of the keystone, abundant and rare taxa (Figure S7). All four taxa groups of prokaryotes showed negative linear relationships with all EMF indices (Figure S7).

**Figure 4:**
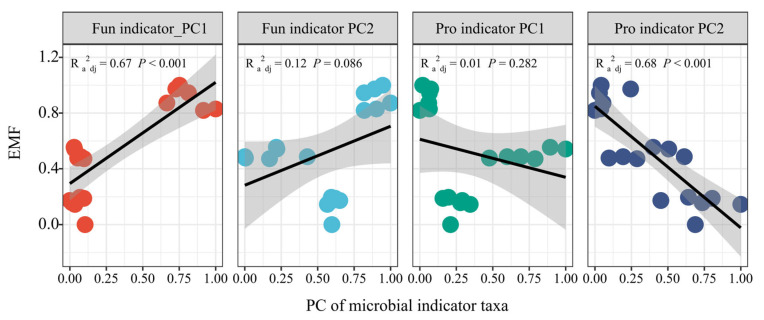
The linear regression of the first two components of microbial indicator taxa and ecosystem multifunctionality. Fun indicator PC1 and Pro indicator PC1 indicate the first components of fungal and prokaryotic indicator taxa, respectively; Fun indicator PC2 and Pro indicator PC2 indicate the second components of fungal and prokaryotic indicator taxa, respectively.

Principal component analysis (PCA) was further used to estimate the whole effects of fungal and prokaryotic indicator taxa on EMF (Tables S3 and S4). The results showed that PC1 of fungal indicators and PC2 of prokaryotic indicators had significant positive (R^2^ = 0.67, *p* < 0.001) and negative linear relationships (R^2^ = 0.68, *p* < 0.001) with EMF, respectively (Figure 4).

### 3.4. Responses of Soil Microbial Activity to Solar Facilities

**Figure 5:**
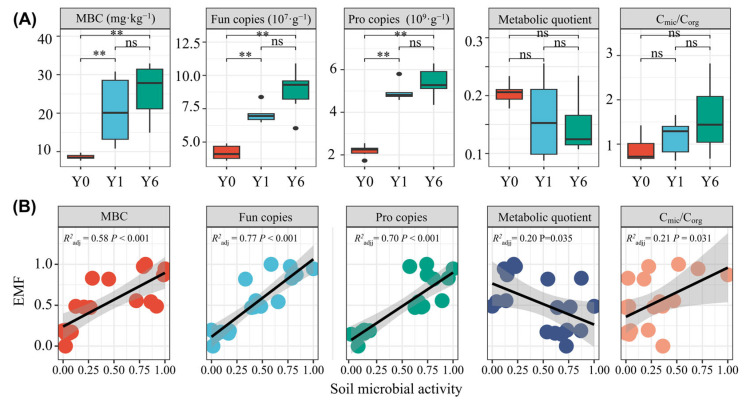
(**A**) The differences in soil microbial activity between solar facility habitats; (**B**) The linear relationships between soil microbial activity and EMF. ns and ** indicate insignificant differences and significant differences at the 0.01 levels, respectively. MBC indicates microbial biomass carbon; Fun copies indicates fungal gene copy number; Pro copies indicates prokaryotic gene copy number. Cmic/Corg indicates the ratio of microbial biomass and soil organic carbon.

Following the installation of the SF, we observed increases in the gene copies of prokaryotes and fungi, microbial biomass carbon and the ratio of microbial biomass and soil organic carbon but a decrease in the metabolic quotient. No significant differences in any of these five indices between Y1 and Y6 were observed (Figure 5A). The linear regression analysis revealed significant positive linear relationships between EMF and all soil microbial activity indices except the metabolic quotient. The soil microbial metabolic quotient had a significant negative linear relationship with EMF (R^2^ = 0.20, *p* = 0.035) (Figure 5B).

### 3.5. Primary Driving Factors of Ecosystem Multifunctionality

**Figure 6:**
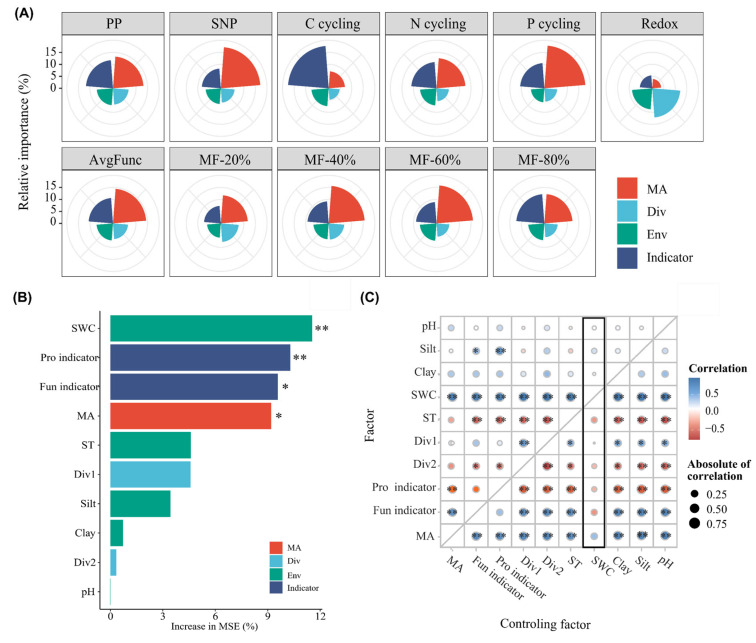
The driving factors and their relative contributions to ecosystem functions and multifunctionality. (**A**) Multiple linear regression of driving factors and ecosystem functions or ecosystem multifunctionality indices. (**B**) Random forest model of driving factors for avgFunc; (**C**) Partial correlation analysis of driving factors for avgFunc. * and ** indicate significant levels at 0.05 and 0.01, respectively.

To determine the primary factors mediating EMF, we assessed the relative importance of environmental factors, biological diversity, microbial indicator taxa and microbial activity. Microbial activity, diversity and microbial indicator taxa were represented by the axles in the principal component analysis (Tables S1–S4). The variance partitioning analysis and multiple linear regression model yielded consistent results, highlighting that microbial activity and microbial indicator taxa had the highest contributions to variations in all aspects of ecosystem function and EMF indices except redox (Figure 6A,B). The random forest model showed that SWC, the Fun indicator, prokaryotic indicator taxa, microbial activity and the soil temperature had the strongest and most significant predictive effects on EMF (Figure 6). The partial correlation analysis further demonstrated that the soil water content, fungal indicator taxa and microbial activity exhibited strong positive correlations with EMF, even after controlling for other factors (Figure 6C).

### 3.6. Regulatory Paths of Eosystem Multifunctionality

**Figure 7:**
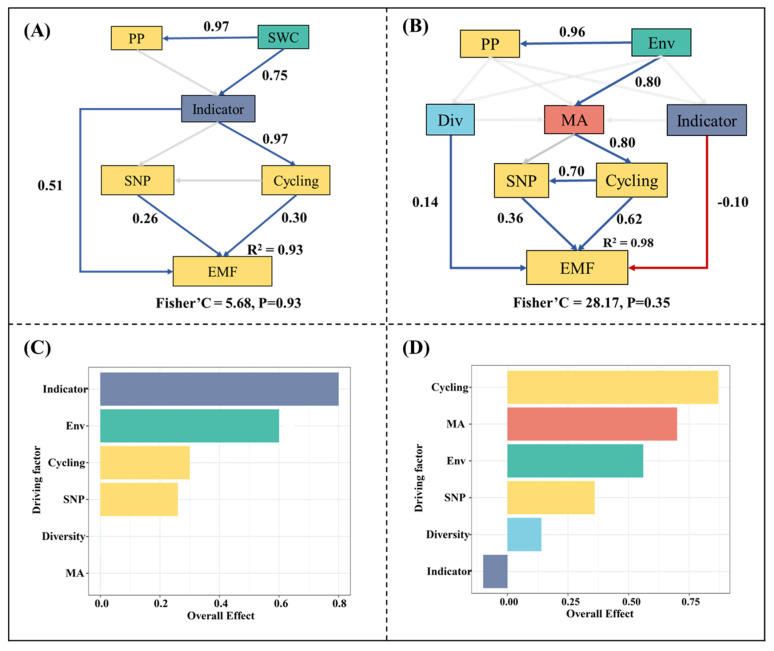
(**A**) Regulatory pathways of ecosystem multifunctionality during the installation period; (**B**) Regulatory pathways of ecosystem multifunctionality during the running period; (**C**) Standardized effects of the primary driving factors on ecosystem multifunctionality during the installation period; (**D**) Standardized effects of the primary driving factors on ecosystem multifunctionality during the running period. The blue, red and grey lines indicate significant positive relationships, significant negative relationships and insignificant relationships, respectively. PP indicates the primary productivity; Env indicates the composite variable of multiple environmental factors. MA indicates the microbial activity. Div indicates the composite variable of the first two principal components of the biodiversity index. Indicator indicates the composite variable of the principal components of microbial indicator taxa. SNP indicates the soil nutrient pool index; Cycling indicates the composite variable of C, N and P cycling.

To facilitate an analysis of the mechanisms and processes regulating EMF, we conducted separate piecewise structure equation model analyses for the installation and running periods. It is worth noting that we retained collinear variables in the model, which can result in excessive standardized coefficients but could effectively represent the ecological causal relationships among the driving factors. In the installation period, microbial indicator taxa were the crucial factor mediating EMF, with a significant direct effect of 0.8 (*p* < 0.001) (Figure 7A). In addition, microbial indicator taxa exhibited significant indirect effects (*p* < 0.05) on EMF by regulating the soil nutrient pool and nutrient cycling functions (Figure 7A and Figure S9). Both the soil nutrient pool and nutrient cycling functions displayed significant positive effects on EMF, with scores of 0.26 and 0.30, respectively (Figure 7A). During the running period, microbial activity, diversity and indicator taxa all exhibited significant effects (*p* < 0.05) on EMF. Despite exerting only indirect effects on EMF, microbial activity exhibited a more substantial overall impact (scoring at 0.68) compared to the other two microbial indicators (Figure S9). In the running period, the soil nutrient pool and nutrient cycling functions also displayed significant positive effects on EMF, but, in contrast to the installation period, they were significantly influenced by microbial activity rather than microbial indicator taxa (Figure 7B).

## 4. Discussion

### 4.1. Solar Facilities Enhance Individual Ecosystem Function and Ecosystem Multifunctionality

In line with our initial hypothesis, individual ecosystem functions and ecosystem multifunctionality (EMF) increased following the installation of the solar facilities (SFs), as shown in Figure 2. This enhancement may be attributed to the conducive environment for plant and microorganism development and growth. Environmental properties, such as the microclimate, soil texture and resource availability, have been shown to influence ecosystem functions [38,39]. Sheltering effects, the interception of shortwave radiation and the regular cleaning of photovoltaic panels promote the soil water content and reductions in soil temperature and evaporation (Figure S10). An increase in soil water content is benefit for plant growth in arid ecosystems, directly promoting primary productivity (Figure S11). The disruptive practices in the SF installation period, and the shifts in temperature, radiation and soil moisture in the SF running period, resulted in changes in the plant community structure, such as significant increases in the abundance of *Leymus chinensis* (Figure S12). *Leymus chinensis* had a relatively higher height and specific leaf area (SLA) compared to other species (Figure S13), which bring higher biomass. Thus, the increased abundance of *Leymus chinensis* could have indirectly promoted primary production (Figure S14). There are significant correlations among primary productivity, the soil nutrient pool and nitrogen and phosphorus cycling (Figure S15), which indicate strong interactions between multiple functions in SF habitats. The increased primary productivity enhances the carbon input to the soil, resulting in higher soil nutrients [40]. The increased substrates and improved water content facilitate the release of soil enzymes, which strengthen soil nutrient cycling functions and further speed up soil carbon, nitrogen and phosphorus turnover [41]. The cooperative effects among multiple functions further result in increased EMF [42]. The piecewise structure equation model indicated that an increase in soil water content promotes microbial activity, enhances the fungal diversity and boosts the abundance of fungal indicator taxa. These factors, in turn, contributed to the promotion of EMF (Figure 7). This result demonstrates the pivotal role of the soil water content in regulating EMF in alpine desert ecosystems [43] and suggests that SFs enhance EMF via a water-induced increase in microbial nutrient cycling [44].

It is necessary to consider the importance of management practices and the types of photovoltaic equipment in impacting ecosystem functions. Management practices can exert profound impacts on the microclimate, soil quality and biological properties [3,45], all of which have the potential to mediate ecosystem functions. In particular, destructive construction activities such as soil leveling, compaction and topsoil removal during SF installation can severely degrade the soil physical structure, reduce the infiltration capacity and lead to the loss of organic carbon. Our observations showed that habitats experiencing both soil leveling and vegetation removal but lacking subsequent ecological restoration exhibited markedly lower soil water content and organic carbon concentrations [5,6]. These soil structural changes directly impair microbial activity and plant growth, undermining the foundation for ecosystem multifunctionality. Therefore, the restoration or enhancement of ecosystem functions through effective post-construction management measures, such as topsoil replacement, soil loosening and reseeding native vegetation, is essential to mitigate these negative impacts and sustain long-term ecosystem functionality in SF habitats.

### 4.2. Primary Driving Factors of Ecosystem Multifunctionality

The results of the variance partitioning analysis, multiple linear regression model, random forest model and partial correlation analysis all revealed that the microbial indicator taxa and microbial activity had higher relative contributions to both individual ecosystem functions and EMF relative to other driving factors (Figure 6 and Figure 7, Figures S8 and S9). These results indicate that microbial indicator taxa and microbial activity may be the primary driving factors mediating EMF in habitats following SF disturbances. Fungal indicator taxa showed significant positive effects on EMF and had the strongest effects among all four types of taxa groups (Figure 4). Fungal indicator taxa have been identified as the most abundant differential taxa in different groups of soil [46]. In our study, they mainly consisted of the phyla *Mortierellomycota, Ascomycota, Basidiomycota* and *Glomeromycota* (Table S5). The SF soil showed higher relative abundances of fungal taxa related to plant growth promotion and soil nutrient transformation, such as the genera *Mortierella* and *Fusidium*. The genus *Mortierella* is responsible for improving access to the bioavailable forms of phosphorus and iron in the soil, the synthesis of phytohormones and 1-aminocyclopropane-1-carboxylate deaminase and the protection of agricultural plants from pathogens [47]. The genus *Fusidium* can prevent pathogens from entering plants by producing antibiotics, such as fusidic acid [48]. We also found the declined relative abundance of fungal pathogens after SF installation, such as the genus *Fusarium*, a soil-borne fungal pathogen [49]. In addition, we found significant positive correlations between most fungal indicator taxa and soil hydrolytic and oxidized enzyme activity (Figure S17). These results indicate that SFs promote EMF via mediating the relative abundance of fungal indicator taxa and associated nutrient cycling functions [50]. The results of fungal functional prediction revealed higher abundances of arbuscular mycorrhizal fungi and lower abundances of animal and plant pathogens in SF soil (Figure S18). This may support the idea that fungi affect EMF by mediating the functional structure [44,51].

Although microbiome data analysis based on relative abundance provides a wealth of information, such as the dynamics of biodiversity and crucial taxa groups for the environmental evaluation of SFs, the profiling of the entire microbial community plays an important and complementary role in the interpretation of ecological aspects [52,53]. We integrated microbial biomass and the metabolic quotient to obtain a profile of the entire microbial community. We found increased microbial activity, as indicated by the declined metabolic quotient and increased C_mic_/C_org_, which could be attributed to the improved environmental properties (Figure S10). Previous studies have indicated that the microbial metabolic activity is influenced by the environmental conditions and substrate quantity and quality [54]; habitats with low substrate quality (i.e., high recalcitrant carbon) [55] and harsh environments like drought could have reduced microbial growth rates and enhanced carbon maintenance costs [56]. The improved soil water content and soil temperature and increased labile carbon substrates, including dissolved organic carbon content, may explain the increased soil microbial metabolic activity following SF installation. The linear regression analysis and structure equation model showed significant positive relationships between microbial activity and EMF (Figure 5B), indicating that SFs can promote EMF by elevating microbial activity. Microbial activity contributed more than diversity and microbial indicator taxa to partial ecosystem functions and EMF indices (Figure 6A,B), indicating that the profile of the microbial community also plays a critical role in governing EMF and specific ecosystem functions.

Taken together, these findings demonstrate that SFs promote EMF primarily by enhancing the soil water content, which in turn increases the abundance of beneficial fungal indicator taxa and improves microbial metabolic activity, driving nutrient cycling and multifunctionality. Beyond these overall patterns, seasonal variations in the SF-induced microclimate may further modulate these effects across the year. During the growing season (May–August), the cooler and wetter microenvironment under SF panels likely alleviates the thermal stress on soil microorganisms, enhancing microbial nutrient cycling and the carbon use efficiency [3]. However, in spring and autumn, reduced photosynthetic activity and lower accumulated growing degree days may constrain primary productivity inputs, potentially limiting nutrient availability for microbial communities [21]. These seasonally distinct responses suggest that the positive effects of SFs on EMF are most pronounced during the growing season, with weaker or negligible impacts in cooler months.

Looking forward, future climate change may further modify these seasonal dynamics. Based on current projections for the Qinghai–Tibet Plateau, rising temperatures, altered precipitation patterns and changes in wind regimes could exert mixed effects on microbial metabolism and EMF. Elevated temperatures may enhance the microbial metabolic rates but could also increase the microbial maintenance respiration costs, potentially reducing the carbon use efficiency over time [56]. Additionally, changes in solar radiation and reduced wind speeds under PV arrays may alter soil moisture regimes, thereby influencing microbial biomass and the community composition. These interactions may either strengthen or weaken the positive effects of SF-induced microclimatic modifications depending on the seasonal context. Further investigations integrating long-term field observations with predictive models are needed to better anticipate how SF-driven and climate-driven factors may jointly regulate EMF in alpine ecosystems.

### 4.3. Distinct Regulatory Paths of Ecosystem Multifunctionality

This study revealed the substantial differences in SFs’ effects on ecosystem functions between the installation and running periods (Figure 2A), which could indicate distinct regulatory mechanisms for EMF during these two periods (Figure 7). During the SF installation period, fungal indicator taxa played a significant role in influencing EMF, both directly and indirectly, by enhancing the overall soil nutrient pool and promoting nutrient cycling processes (Figure 2A). This can be primarily attributed to the relative abundance growth of these taxa during the installation period. Fungal indicator taxa exhibited strong correlations with environmental factors (Figure S19); the substantial changes in the environmental conditions following SF installation led to variations in the relative abundance of these taxa. Given that most fungal indicator taxa showed significant positive correlations with soil enzyme activity related to soil carbon, nitrogen and phosphorus nutrient cycling (Figure S17), the increased relative abundance of these taxa could enhance nutrient cycling functions and increase the soil nutrient content, which further promote EMF.

In addition, we found substantial shifts in the abundance of arbuscular mycorrhizal fungi and pathogenic fungi in the initial installation stage (Figure S18), which have been reported to have remarkable correlations with EMF [57,58]. The shifted abundance of arbuscular mycorrhizal fungi and pathogens may also contribute to the growth of EMF. In contrast to the initial phase, the relative abundance of fungal indicator taxa, arbuscular mycorrhizal fungi and pathogenic fungi remained relatively steady throughout the running period (Figure S18), which may indicate the decreased impact of taxa on EMF. Microbial activity had the most significant overall impact on EMF, with fungal indicator taxa contributing only minimally (scored at 0.1) (Figure 7B). These results demonstrate the reduced importance of fungal taxa and the dominant role of microbial activity in mediating EMF during the running period. Moreover, both the soil nutrient pool and nutrient cycling were directly influenced by microbial activity during the running period, which further underscores the indirect regulating effects of microbial activity on EMF (Figure 7B). These findings suggest a shift in the primary influencing factors of EMF, transitioning from specific microbial taxa groups during the installation period to microbial activity during the constant running period.

### 4.4. Research Implications

This study focused on variations in EMF across different operational stages of solar facilities and identified the primary biotic and abiotic drivers regulating these changes. These findings provide valuable guidance for improvements in environmental monitoring frameworks and could inform management practices for SFs in arid ecosystems. Specifically, two aspects should be prioritized: (i) establishing sensitive biological indicators, such as key fungal taxa (*Mortierella* and *Fusidium* genera), for targeted ecological monitoring [50], and (ii) identifying microbial activity as early-warning indicators of potential ecosystem function shifts during long-term operation. These insights could support the development of precision management strategies in SF habitats.

Beyond the scope of ecological monitoring, our results also have implications for broader land use planning, particularly the potential to integrate SFs with agricultural practices such as agrivoltaics. The observed microclimatic changes, including reduced air temperatures, increased soil moisture and moderated environmental stress under solar panels, suggest that SFs could create localized niches favorable for certain drought-adapted crops or for improved forage availability in otherwise marginal grazing lands [57]. In arid alpine regions, potential candidates for integration may include hardy perennial grasses (e.g., *Leymus chinensis*) and drought-tolerant forage species suitable for extensive livestock grazing systems, such as sheep or yak herding. However, the diminishing marginal effect that we observed, where the ecosystem function gains decreased over time, indicates that integrating photovoltaic infrastructure with agricultural or pastoral systems will require active management, particularly in terms of soil water retention, drainage control and plant species selection to maintain the productivity benefits.

Additionally, enhanced soil microbial multifunctionality under SFs could contribute to long-term soil fertility improvement, supporting broader goals of ecological restoration in degraded arid regions. Given the increasing global expansion of industrial-scale solar facilities, establishing long-term ecological monitoring systems and integrating adaptive land management approaches will be essential in balancing renewable energy development with local ecological sustainability and livelihood security [6].

## 5. Conclusions

Our study demonstrated that solar facility installation significantly enhanced ecosystem multifunctionality in arid alpine grasslands, with a markedly stronger effect during the installation period (31.4%) compared to the running period (3.5%). This enhancement was primarily driven by improved soil water content, which stimulated soil microbial processes. Importantly, we identified a distinct shift in the underlying mechanisms regulating ecosystem multifunctionality: during the installation period, the enhancement was mainly associated with changes in fungal indicator taxa that promoted nutrient cycling, whereas, in the running period, microbial activity became the dominant driver sustaining EMF. These findings highlight that solar facilities not only modify ecosystem functions through physical disturbances but also induce lasting biotic responses mediated by soil microbiomes. Given the single-season scope of this study, future multi-year and multi-season research is needed to fully understand the long-term ecological sustainability of solar facilities in fragile arid ecosystems.

## Figures and Tables

**Table 1:** The potential driving factors of ecosystem multifunctionality.

Category	Group	Driving Factor
Biotic factor	Microbial activity (MA)	Gene copy number of fungi
		Gene copy number of prokaryotes
		Microbial biomass carbon
		Microbial metabolic quotient
		Ratio of microbial carbon to organic carbon
	Diversity (Div)	Taxonomic diversity of plants
		Taxonomic diversity of fungi
		Taxonomic diversity of prokaryotes
		Phylogenetic diversity of plants
		Phylogenetic diversity of fungi
		Phylogenetic diversity of prokaryotes
		Functional diversity of plants
		Functional diversity of fungi
		Functional diversity of prokaryotes
	Indicator taxa (Indicator)	Indicator taxa of fungi
		Indicator taxa of prokaryotes
Abiotic factor	Environmental factor (Env)	Evaporation
		Soil temperature
		Soil water content
		Soil pH
		Clay
		Silt
		Sand

## Data Availability

The data presented in this study are available upon request from the corresponding author. The data are not publicly available due to privacy restrictions.

## Supplementary Materials

The following supporting information can be downloaded at: https://www.mdpi.com/article/10.3390/microorganisms13071464/s1. Table S1: The PCA of microbial activity. Table S2: The PCA of different alpha diversity index. Table S3: The PCA of fungal indicator taxa. Table S4: The PCA of prokaryotic indicator taxa. Table S5: The linear regression coefficient of fungi indicator taxa on ecosystem multifunctionality. Figure S1 The remote images of various environmental factors (A) among sites and the significant analysis among groups (B) before solar park installation. Figure S2: The correlations between plant functional indicators. Forks indicate a insignificant correlations at 0.05 level. Figure S3: The significant differences in ecosystem functioning index. Figure S4: The significant differences in ecosystem functioning categories. Figure S5: The significant differences in EMF index. Figure S6: The effects of solar parks on alpha diversity. Figure S7: The standard linear effects (mean ± 95% confidence intervals) of the relative abundances of difference types of taxa of prokaryotes (A) and fungi (B) on EMF. Figure S8: The linear relationships between microbial activity and EMF. Figure S9: The linear relationships between microbial activity and ecosystem functions. Figure S10: The significant differences in environmental factors between treats. Figure S11: The correlations of environmental factors and ecosystem functioning and multifunctionality. Figure S12: The changes in the relative abundance of plant species. Figure S13: The differences in community-weighted mean of plant height and SLA. Figure S14: The standard regression coefficients of plant traits on ecosystem functioning and multifunctionality indices. Figure S15: The correlations among ecosystem functioning and EMF. Figure S16: The correlations between α-diversity indices of plant, prokaryote and fungi. Figure S17: The correlations between the relative abundance of fungal indicator taxa and soil enzyme activities. Figure S18: The changes in the relative abundance of fungal guilds. Figure S19: The correlations between environmental factors and relative abundance of fungal indicator taxa. References [59,60,61,62,63] are cited in the Supplementary Materials.
