# Supplementary material for: Ecosystem Multifunctionality Regulated by Soil Microbial Activity and Indicator Taxa Versus Biodiversity for Industrial Solar Facilities on the Qinghai–Tibet Plateau
Source: Microorganisms. 2025 Jun 24;13(7):1464. doi: 10.3390/microorganisms13071464 (PMC12298980; doi:10.3390/microorganisms13071464)
Supplement: Supplementary file 1 [file microorganisms-13-01464-s001.zip › microorganisms-3654189-supplementary.pdf]

Supplemental methods:

GIS analysis:

Topographic diversity (TD) was estimate based on images from “Global ALOS Topographic Diversity”; Mean annual precipitation (MAP) and mean annual temperature (MAT) were estimated using images from “WorldClim Climatology V1” dataset. Soil temperature (ST) was measured with images from “USGS Landsat 7 Level 2, Collection 2, Tier 1” dataset; Aboveground carbon density (ACD) and belowground density (BCD) was calculated using images from “Global Aboveground and Belowground Biomass Carbon Density Maps”; Global primary productivity (GPP) and Evaporation (ET) were calculated with images from “PML_V2 0.1.7: Coupled Evapotranspiration and Gross Primary” dataset; NDVI and EVI data were from “MOD13Q1.061 Terra Vegetation Indices 16-Day Global 250m” dataset;

All images were obtained, calculated and plot with Google Earth Engine. Only images before 2013 (The initial solar park installation) were obtained. Except for TD, ACD and BCD, all indicators were represented by the mean values over a ten-year period, specifically from 2013. ACD and BCD used the image from 2010. TD used image from 2006-2011.The values of all indicators of all sample site were extracted according to their geographic location. The significant differences between groups were calculated using ANOVA in R4.0.3.

The analysis of individual ecosystem functions:

The aboveground and belowground partition of plant was oven-dried at 75 °C for 48 h to calculate the aboveground biomass, belowground biomass. Soil organic carbon was measured with the $K_2Cr_2O_7$ oxidation-reduction titration, soil total nitrogen was determined with the Kjeldahl digestion methods. Soil total phosphorus and total potassium were calculated by flame photometry method after melting with sodium hydroxide (Dick and Tabatabai,1977). Dissolved organic carbon were determined using TOC analyzer (TOC-L CPN, Shimadzu, Japan). The soil ammonium nitrogen, nitrate nitrogen and available phosphorus was obtained from K_2SO_4 using colorimetric assays (Delgado-Baquerizo, et al.,2013). The soil available potassium was calculated using flame photometric protocol by 1 mol/L NH4OAc extraction. The carbon mineralization rate was measured using Alkali absorption method after a two-week incubation at 25℃ and 60% soil water content (Bridgham and Ye,2013). Nitrification and ammonification represented the measurements of the discrepancy in NH4+-N and NO3--N levels between the original natural soil and soil that underwent a two-week incubation at 25°C with 60% soil water content. Nitrogen mineralization was calculated as the combined sum of Nitrification and ammonification. The soil hydrolytic enzyme activities including alpha-glucosidase (αg), beta-glucosidase (βg), beta-xylosidase, leucine aminopeptidase, N-acetyl-β-D-glucosaminidase, and alpha-galactosidase were measured using microplate fluorimetric approaches (Marx, et al., 2001). The soil oxidative enzyme activities (peroxidase and polyphenol oxidase) were measured using microplate protocols with substrates of l-3,4-dihydroxyphenylalanine (l-DOPA) (Bach, et al., 2013).

Supplemental tables and figures:

Table S1 The PCA of microbial activity

Indicator	PC1
MBC	0.979
Fun copies	0.874
Pro copies	0.794
metabolic quotient	-0.828
C_{mic}/C_{org}	0.8
Proportion Var	73.60%

Table S2 The PCA of different alpha diversity index

Taxa	Aspect of α diversity	PC1	PC2
Vegetation	Species richness	0.661	-0.273
	Phylogenetic diversity	0.22	-0.66
	Functional diversity	0.704	-0.351
Prokaryotic	Species richness	0.867	-0.227
	Phylogenetic diversity	0.839	-0.266
	Functional diversity	0.796	0.174
Fungi	Species richness	-0.134	0.905
	Phylogenetic diversity	-0.281	0.872
	Functional diversity		0.886
Proportion of variance		35.2%	35.2%
Cumulative variance		35.2%	70.2%

Table S3 The PCA of fungal indicator taxa

Fungi ASV	PC1	PC2
de5802e47a99805dcc75067245e01343	0.893	0.318
41f4e088dbe0a6e7e032b0343e0754f8	0.8	
814b01f9295131de35b27565c2741f7a	-0.347	-0.241
98538960e0f38359a2022b8b8a476b32	-0.356	-0.235
7714ff67547eaf595067cc338c4c633e	0.878	
2478d9c43912c93848e329426aa12347	0.862	0.179
6229f56063f818a9cc9944bc2aaf65d7	0.897	0.411
6dcff1b9ab2f5c6e5026d7d99b9d8e6a	0.881	0.447
e1fc8ba886852b69d053f6a76f766538	0.92	
a3bad9179e92fb86710674c3f140f3de	0.944	0.254
17c72b50357d21ac60cac60a339b6b40	0.925	
971258c78690ac34f6ba622d42b33284	0.733	0.305
1fbf6bd19328f30c9143e96162500621	0.75	0.406
4f982847195ab67f4210df0afaf75069	0.748	0.14
4e1eb31df2fa573b450d6112f07c4f90	0.763	0.603
1c10793017f254edb9789f70cc634d5b	0.839	0.328
490eb2fa86b0cb3085ab097b7ff776b3	0.648	0.281
3b406c0860eb2c87f3bfc35821d8abc4	0.321	0.618
bfbc263285c530535e8a7967cbffcd40	0.605	0.548
9a7a2cde7eb5dd711c90d173999acc2a	0.752	0.3
2b75a35270e9a54bdba8ed85b09708ce	0.419	0.584
1135ae45776aebbb94869af1b55a9885	0.573	0.778
5df88103cdf16b5d87d5749015f1a9fe	-0.324	-0.185
3f7386ea62aa1702c8d8242024a16688	0.651	0.31
66c033e300a8bb08fb1b8c51b0b3e7f5	0.797	
34b42a421c48da24ee94c92e836bf958	0.281	0.771
25353abf2d0e6c28318793b3f6fc069c	0.173	0.858
d4691b848cad5fc1c3b1bc43b807ee3d	0.95	
71407a7bd7b77477a130245f381287b5	0.887	
bb82e381dc58dac2ad890f4c053a898d	0.421	0.741
1ec37fc56fd088decfcd77a4ce6b3f23	0.702	0.409
7ebaf8df21e204dff62b2e0d0a524dc1	0.622	
7afeea0385a20bc367e184c71bc94d84	0.602	0.351
db9f027f24f5b0592688adeda3fbb396	0.846	0.194
2e08478590988bab33e00f343aecf984	0.805	0.134
9557c6054e35dafb56bbe0b607f69ec1	0.665	0.277
Proportion of variance	42.3%	24.8%
Cumulative variance	42.3%	67.1%

Table S4 The PCA of prokaryotic indicator taxa

ASV	PC1	PC2
0b9e7b1532d86d28d872bfd0ba38a575	0.64	0.48
427fc0960b6842d26c1215ca3ec610b8	0.33	0.73
0018939bf02c32c9a63c42ade52cefad	0.76	0.02
1a56ad3ec2b87cfbeaba8976be08b4b9	-0.22	-0.74
5cf37e80516a615f77bd5e97f18ae417	0.87	0.20
e0aa49e308e34c6e69442514d947224c	0.46	0.78
d63e3ee111f0d41a61942a4d040b4d1a	0.53	0.72
5ecc7a81f01666876c72ebdd7d7abe71	0.27	0.77
7483b49fd3c2e40e8f02f09a4ac8c83d	-0.06	-0.74
66a395fbf90e077857cc54d8b1d65bd0	0.75	0.52
050a60004d05100116598fc38d510709	0.84	0.26
bb36622fd7c17ea45e82629c3beef829	0.70	0.52
d424c77359d10dbf4183f4d5a97a077b	0.69	0.53
1e5252f7f059d825508ebf3880dffd92	-0.24	-0.57
ee4c323e8bb93997f0b8bcb9a5efddb0	0.28	0.75
b9d3e3d87e83dc69a1f986a657b17675	0.78	0.13
99be8709ae7b069e466beca6bdaa36f4	0.78	0.16
f99ccaaf35afbf4166f84ccb6c357d31	0.14	0.82
2e33ef76544ec5faec4cfb3b5e0635f6	0.20	0.78
e5b8aa4e9ca9b255a6e1e7ea5f0c576e	0.84	-0.15
cd7c4bed24d7fb29078e9c127a4fa72e	0.34	0.69
5533a6eeeda6e26e35b99f038db2c1b0	0.31	0.71
95e7e390c3cdb823bd6aef8cae5786d1	0.88	0.25
c31a8652dd26b703c53b5e11ccaadc27	0.68	0.56
f3bb89b490e8a3194866d1250f10bf53	0.84	0.31
bffd6e7320d0aa746feb22974f1388eb	0.77	0.40
252ba0276b58c6239fcabd31c6082a06	0.77	0.30
83a8d78991bd4ac8893514b669755979	0.90	0.29
af472e0465ab3f8befd83022325d6bee	0.61	0.64
e9a5b8cb54082a97f33699b0ed03e004	0.78	0.34
33def3d34332c81ea23baf6a107cec2f	0.85	0.21
7997386ba2220d045f0c4c8d2070d451	0.57	0.66
e0e0a3865c878986a3a82124db7bbfc9	0.78	0.43
a741863e09571d053758348365a1764b	0.88	0.22
2aa2553127e47c34d6fbb3f7a4eb8d97	0.85	0.30
0fe5dbc099d90ca7e8a7cfb7ef5b7dda	0.76	0.23
9cf2c07188b781c65d6db92c183ee7e2	0.00	-0.79
9f36e716dc795cadde3cbb417c0b58a7	0.56	0.65
2f42cb97bb022654970612cc51be7ec0	-0.01	-0.72
5b532697a7f4e5421705e80f75115f1a	-0.15	-0.56
9ebd4d47c25e82ab5fff6ca85f1ad499	0.70	0.59
f43b95d7b10a6e1c9a52c7b4c23ad406	0.54	0.51
7d827b0b29a5a837e7e6befd44622855	0.35	0.68
205e9006842d47d1a2d1caf6a96e0c88	0.66	0.58
dcb52c0fa70be1c1498e23a109e2c1f3	0.85	0.43
67162cb636e12540f0c552c844957f16	0.17	0.75
03ad73297cdda574a9a28a75a1ddec42	0.67	0.53

5f3b1ac1bad61b46c239460b04a5e9b3	-0.08	-0.74
46d3edd4963d678026af728de6f7ceba	0.31	0.73
73a53dc6ee8da862815c12c10d21b7c2	0.03	-0.78
9eb0cfbe5edb1d5322cb67fccc4dbb7d	-0.02	-0.81
970bb55d0fc4c3ab5d87abec40d16ac2	0.69	0.44
2cf7b66a7aeab616b159ed46a9b762a9	0.79	0.12
3c9d8c15d5d81ecdcbea3147574dc2be	-0.32	-0.84
f8fdaa28dfcf388e0a508e350cd038c3	0.00	-0.78
5b2ec716f65953e849cafba47fc42a95	0.89	0.27
677977d032376dc5d016db6d12c8b3b7	-0.31	-0.60
cc67fd6eead1b6cceabd3237fe6c0f54	-0.10	-0.73
48ae2486d3915aaffb15121e5fc7c424	-0.27	-0.77
a8c0803132cb976086dab0876bd2092a	0.64	0.56
54607d0315f20241270cf07be00d9765	0.90	0.27
fc87241c6abb3659c660ebd3a1f239b2	-0.23	-0.72
e27c3062c3ebfd305447410c7c7ca1ea	0.48	0.70
1391dc70b0b9bea6bf3803af91b54cb2	0.81	0.02
f3eb1f5aadb0a883dba2317cfe1371c9	0.54	0.66
ae220bec5547084689b9f04ace6ffa37	0.90	0.10
c56f0a37bfaf72d158edc6f7443618a6	0.61	0.33
c02da5a9e3cb8a42c9bf3979787039d2	0.79	0.32
6640f8ec6902ed1fd5837869a6054fbe	0.62	-0.03
becc1d062407954300656d298b73a3bd	0.93	0.21
19ae9738a33b9cd82c8e72b8d463bf0c	0.91	0.03
4a2bd0d5b339798ac5fac87d0cce30e8	0.92	0.16
af62728cdc8b3e916fabf7809066bf28	0.74	0.18
c7a2ebc126c24dfda0789e4b836ccfe3	0.86	0.38
cfbbf3ba7281dc4603525c562e10d508	-0.23	-0.56
e6633f62777f58f902a3cd0c50ea2b36	0.88	-0.04
79be0d60434a3d801cf4cb920b550a53	0.54	0.58
f2c712672534179880dc3bdf0410a163	-0.19	-0.88
9c0e85f09a1fb8e47ef2720b9d4c907c	0.83	0.30
1fd7cc43550070fed3fd6226cf8918e3	0.88	0.05
26fe671d01f87cf271df993c7231c6a2	0.01	-0.71
5953bd472aebc5aa8c9b1a190c261260	0.76	0.21
7b32f308dddb1b981af50da2c6e9aad 9	-0.17	-0.83
439c16f179139295354d8e7e8e880ea6	0.85	0.17
6c6b6195518dc76aae15b2e48537c2c9	-0.20	-0.75
f6edbcb91f18c1dcc550ce2c68333165	0.87	0.18
619ca40edde3745255e205747c3fc8a3	0.75	0.47
e5f9b3828852940fd35d30a2792d84f9	-0.01	-0.74
03eb4945b665c95f00f9208c2b849bcc	0.54	0.50
9c2aae357c9f710a794e3c7ec0b0cb74	0.77	0.13
26074b3c2343a3e408ed81c095c5b376	0.64	0.28
e8a499c569880b49145fc0bb9a2f71c5	-0.03	-0.64
21df09abf781000e60634463fbec6f66	0.79	-0.07
eb76da56514e4de6474fe5b6be63ca5c	0.66	0.47
da5500b20ee2f059c9e1832a9ca626ce	0.80	0.30
Proportion Var	39.6%	28.6%

Cumulative Var	39.6%	68.2%

Table S5 The linear regression coefficient of fungi indicator taxa on ecosystem multifunctionality (AvgFunc)

ASV	Kingdom	Phylum	Estimate	Pr(>|t|)
de5802e47a99805dcc75067245e01343	Fungi	Mortierellomycota	0.28	0.00
41f4e088dbe0a6e7e032b0343e0754f8	Fungi	Ascomycota	0.28	0.02
814b01f9295131de35b27565c2741f7a	Fungi	Ascomycota	-0.33	0.00
98538960e0f38359a2022b8b8a476b32	Fungi	Ascomycota	-0.25	0.00
7714ff67547eaf595067cc338c4c633e	Fungi	Ascomycota	0.23	0.02
2478d9c43912c93848e329426aa12347	Fungi	Ascomycota	0.32	0.01
6229f56063f818a9cc9944bc2aaf65d7	Fungi	Basidiomycota	0.30	0.00
6dcff1b9ab2f5c6e5026d7d99b9d8e6a	Fungi	Basidiomycota	0.32	0.00
e1fc8ba886852b69d053f6a76f766538	Fungi	Ascomycota	0.27	0.02
a3bad9179e92fb86710674c3f140f3de	Fungi	Basidiomycota	0.32	0.00
17c72b50357d21ac60cac60a339b6b40	Fungi	Ascomycota	0.26	0.02
971258c78690ac34f6ba622d42b33284	Fungi	Basidiomycota	0.38	0.00
1fbf6bd19328f30c9143e96162500621	Fungi	Ascomycota	0.27	0.00
4f982847195ab67f4210df0afaf75069	Fungi	Ascomycota	0.34	0.00
4e1eb31df2fa573b450d6112f07c4f90	Fungi	Ascomycota	0.29	0.00
1c10793017f254edb9789f70cc634d5b	Fungi	Ascomycota	0.35	0.00
490eb2fa86b0cb3085ab097b7ff776b3	Fungi	Ascomycota	0.23	0.04
3b406c0860eb2c87f3bfc35821d8abc4	Fungi	Ascomycota	0.32	0.02
bfbc263285c530535e8a7967cbffcd40	Fungi	Basidiomycota	0.28	0.00
9a7a2cde7eb5dd711c90d173999acc2a	Fungi	Basidiomycota	0.38	0.00
2b75a35270e9a54bdba8ed85b09708ce	Fungi	Ascomycota	0.31	0.00
1135ae45776aebbb94869af1b55a9885	Fungi	Ascomycota	0.28	0.00
5df88103cdf16b5d87d5749015f1a9fe	Fungi	Ascomycota	-0.31	0.02
3f7386ea62aa1702c8d8242024a16688	Fungi	Ascomycota	0.30	0.02
66c033e300a8bb08fb1b8c51b0b3e7f5	Fungi	Basidiomycota	0.27	0.04
34b42a421c48da24ee94c92e836bf958	Fungi	Ascomycota	0.33	0.00
25353abf2d0e6c28318793b3f6fc069c	Fungi	Basidiomycota	0.32	0.01
d4691b848cad5fc1c3b1bc43b807ee3d	Fungi	Ascomycota	0.30	0.01
71407a7bd7b77477a130245f381287b5	Fungi	Ascomycota	0.29	0.03
bb82e381dc58dac2ad890f4c053a898d	Fungi	Ascomycota	0.27	0.00
1ec37fc56fd088decfcd77a4ce6b3f23	Fungi	Basidiomycota	0.32	0.00
7ebaf8df21e204dff62b2e0d0a524dc1	Fungi	Ascomycota	0.30	0.02
7afeea0385a20bc367e184c71bc94d84	Fungi	Glomeromycota	0.31	0.00
db9f027f24f5b0592688adeda3fbb396	Fungi	Ascomycota	0.33	0.00
2e08478590988bab33e00f343aecf984	Fungi	unidentified	0.30	0.01
9557c6054e35dafb56bbe0b607f69ec1	Fungi	unidentified	0.21	0.02

Figure. S1 The remote images of various environmental factors (A) among sites and the significant analysis among groups (B) before solar park installation.

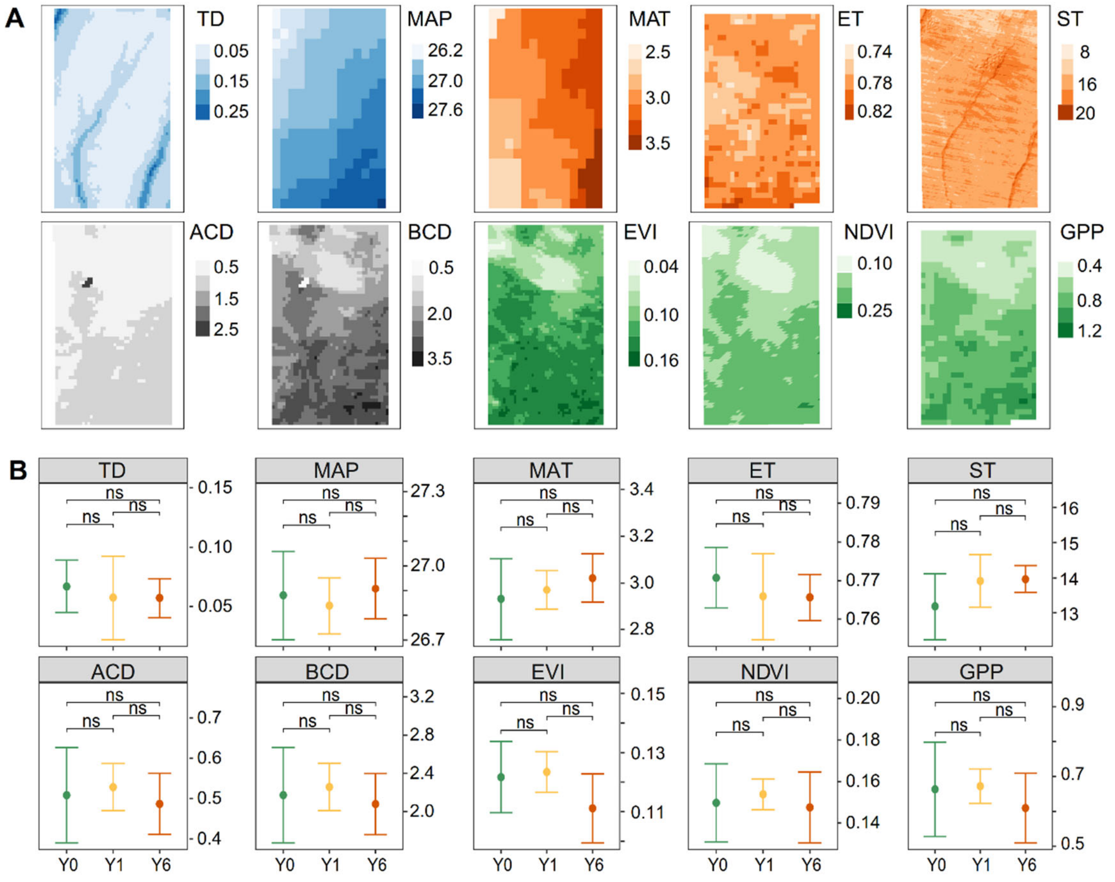

Figure. S2 The correaltions between plant functional indicators. Forks indicate a insignificant correlations at 0.05 level.

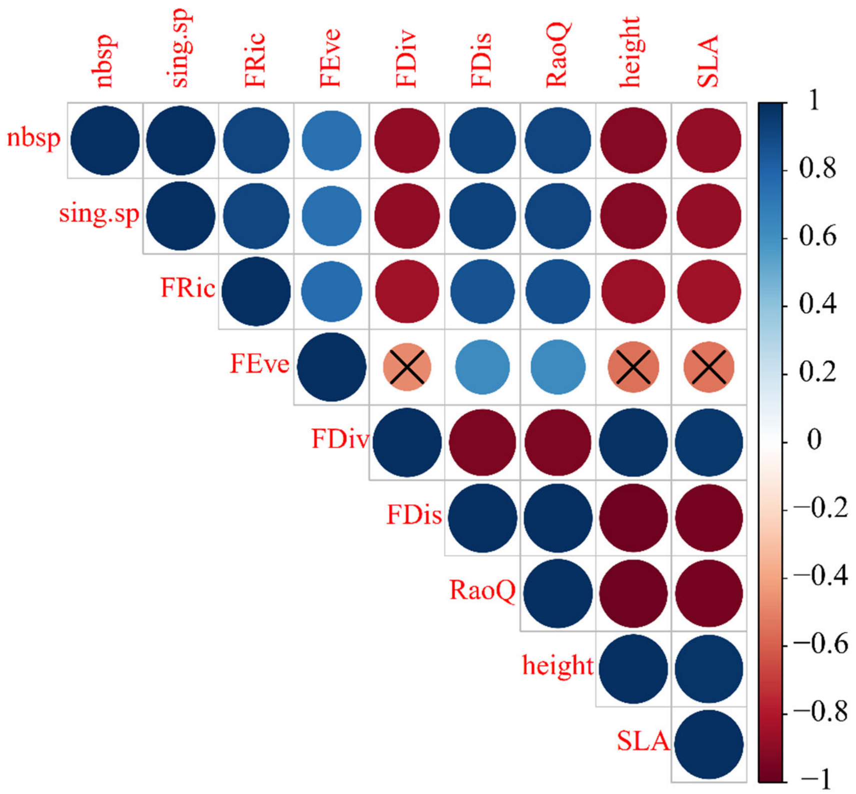

Figure. S3 The significant differences in ecosystem functioning index. ns, * and ** indicate insignificant difference, significant difference at 0.05 level and significant difference at 0.01 level.

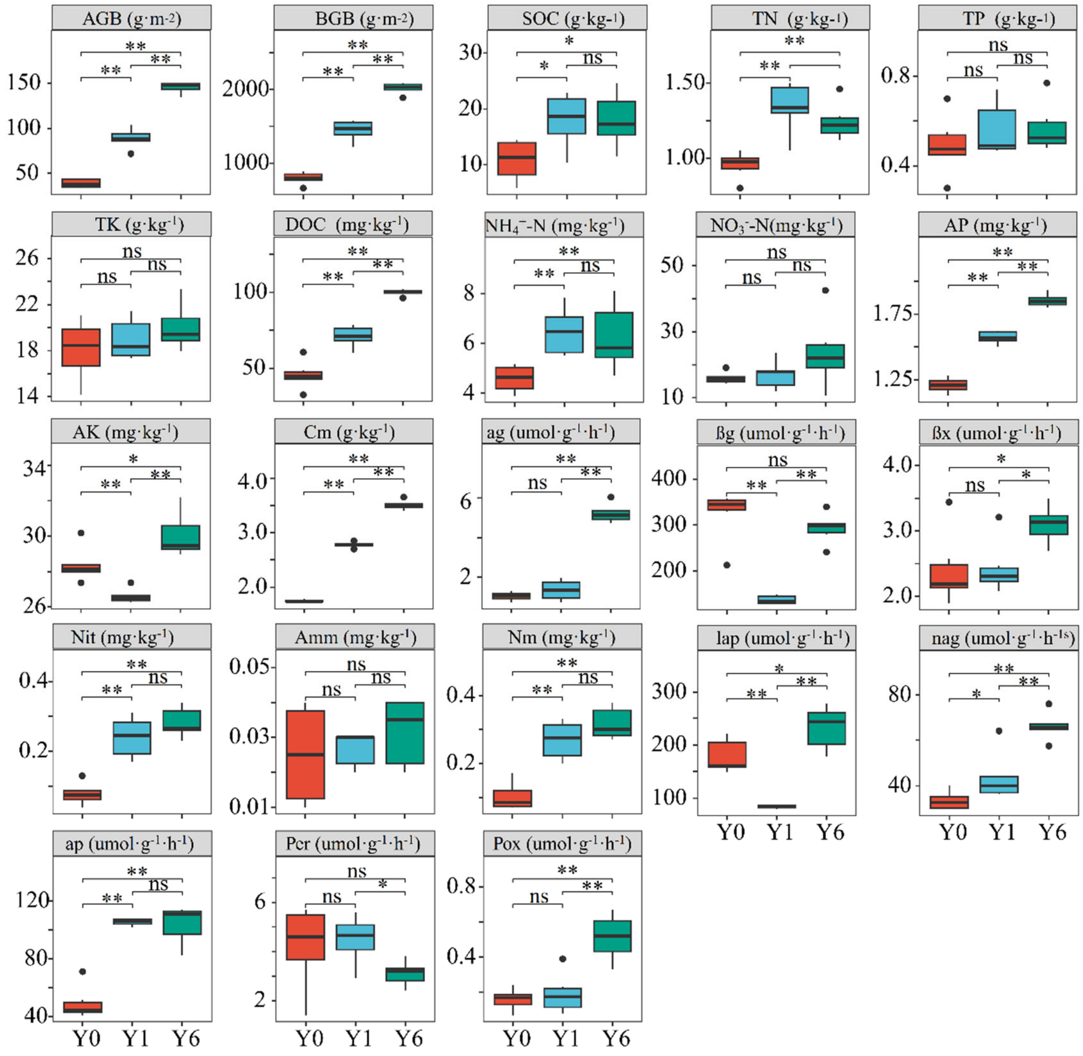

Figure. S4 The significant differences in ecosystem functioning categories. ns, * and ** indicate insignificant difference, significant difference at 0.05 level and significant difference at 0.01 level.

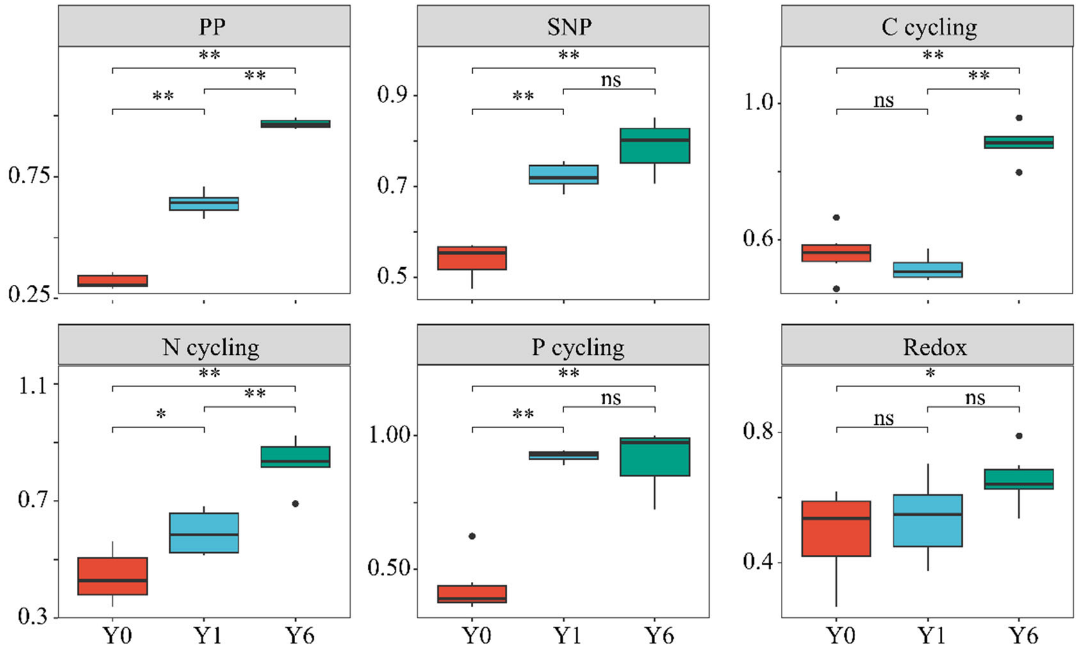

Figure. S5 The significant differences in EMF index. ns, * and ** indicate insignificant difference, significant difference at 0.05 level and significant difference at 0.01 level.

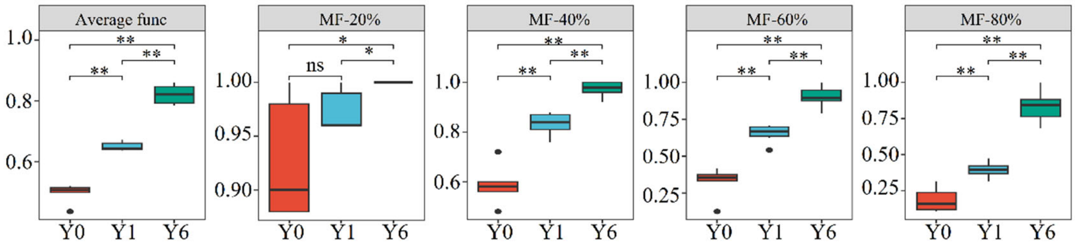

Figure. S6 The effects of solar parks on alpha diversity

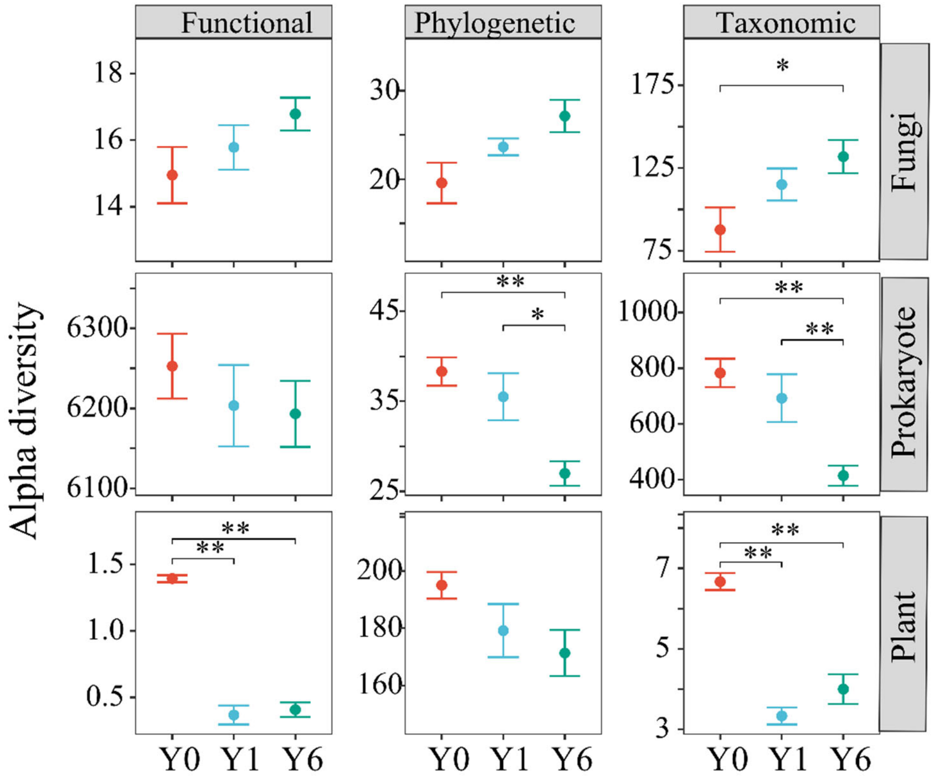

Figure. S7 The standard linear effects (mean ± 95% confidence intervals) of the relative abundances of difference types of taxa of prokaryotes (A) and fungi (B) on EMF. Whole indicate the whole dataset.

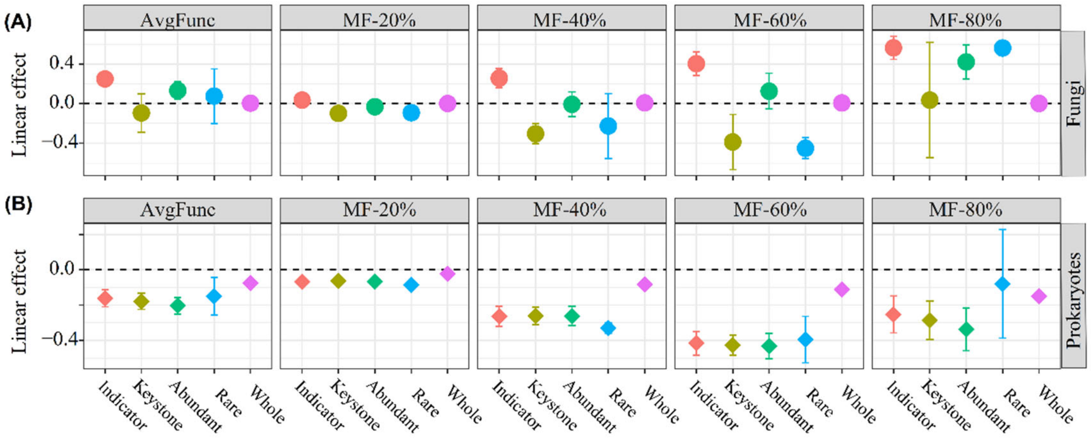

Figure. S8 The linear relationships between microbial activity and EMF

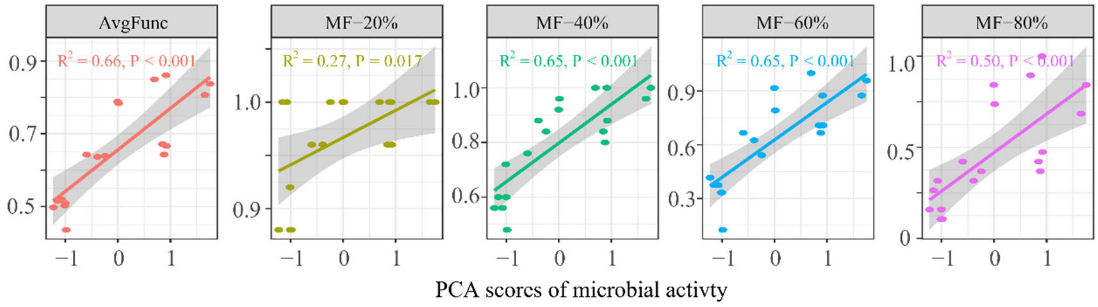

Figure. S9 The linear relationships between microbial activity and ecosystem functions

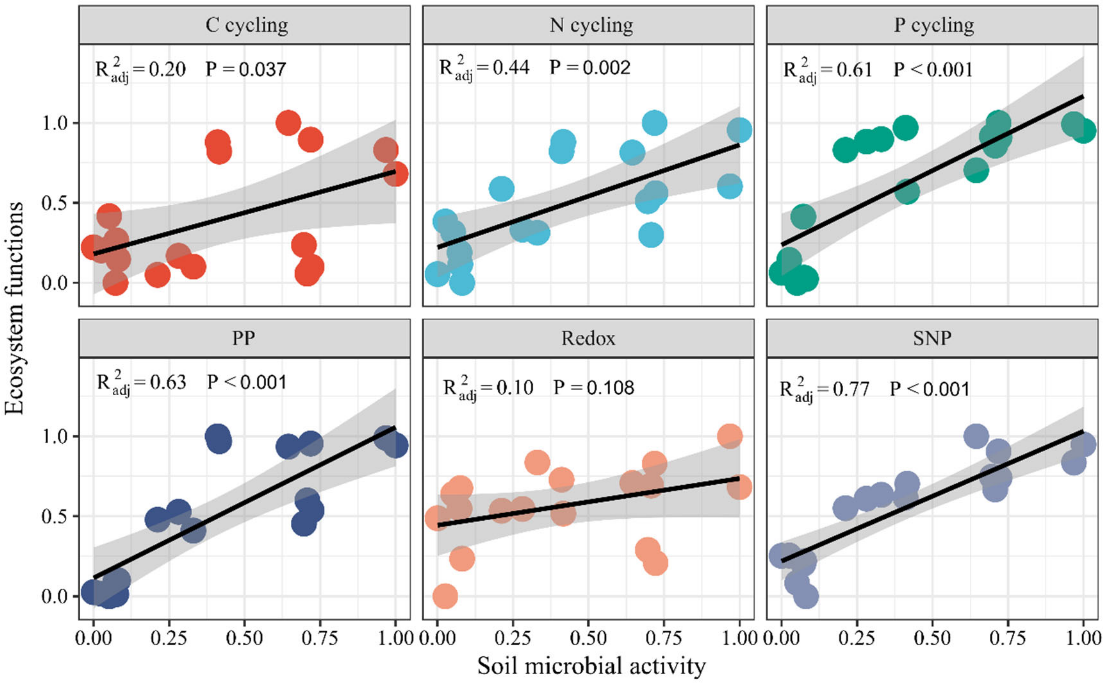

Figure. S10 The significant differences in environmental factors between treats. ns, * and ** indicate insignificant differences, significant differences at 0.05 level and significant differences at 0.01 level.

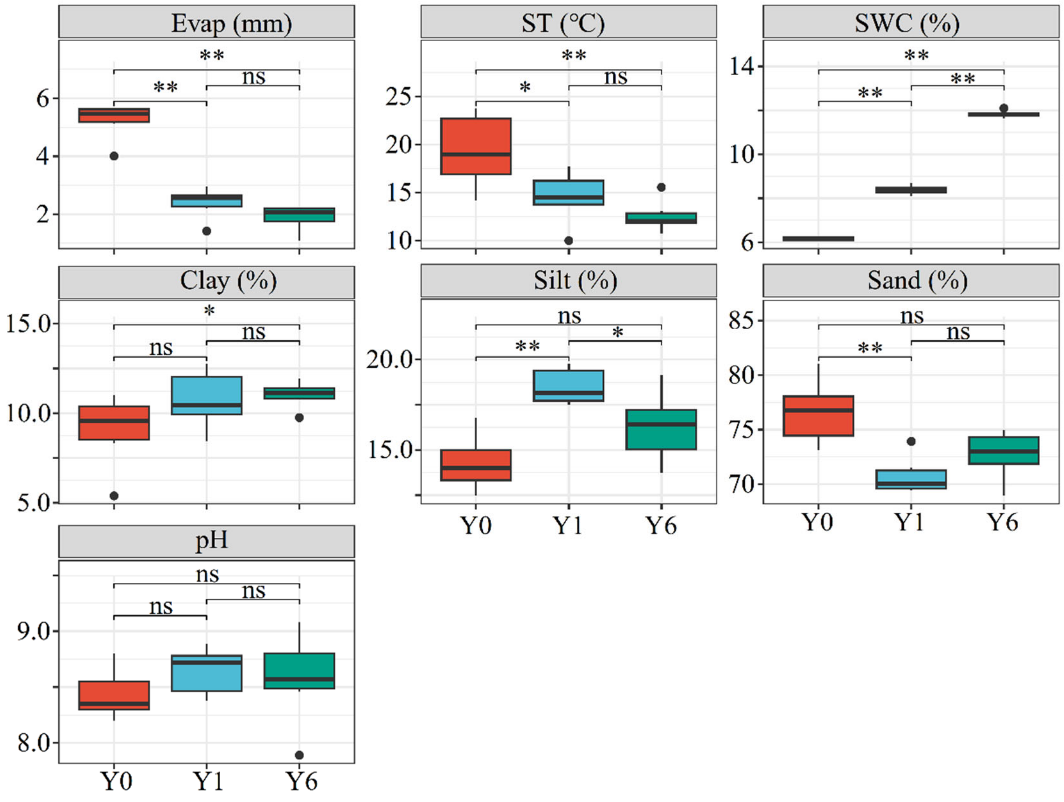

Figure. S11 The correlations of environmental factors and ecosystem functioning and multifunctionality. * indicates significant correlations at 0.05 level.

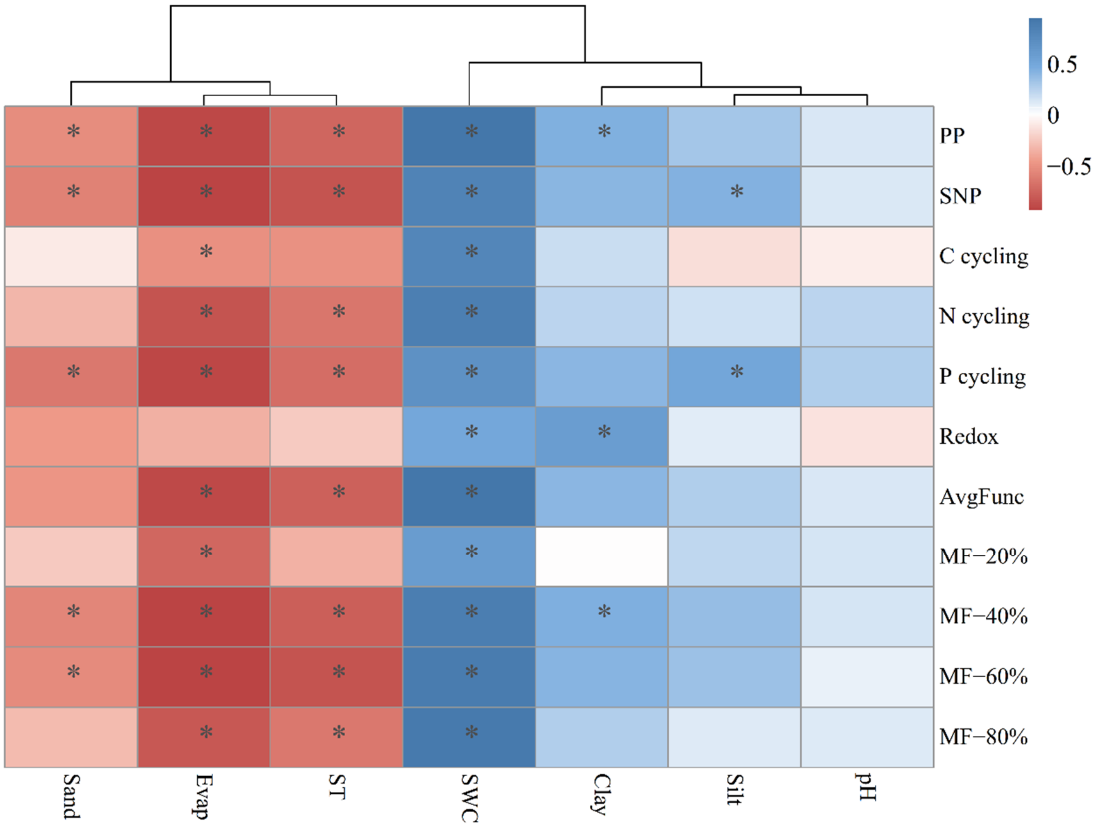

Figure. S12 The changes in the relative abundance of plant species

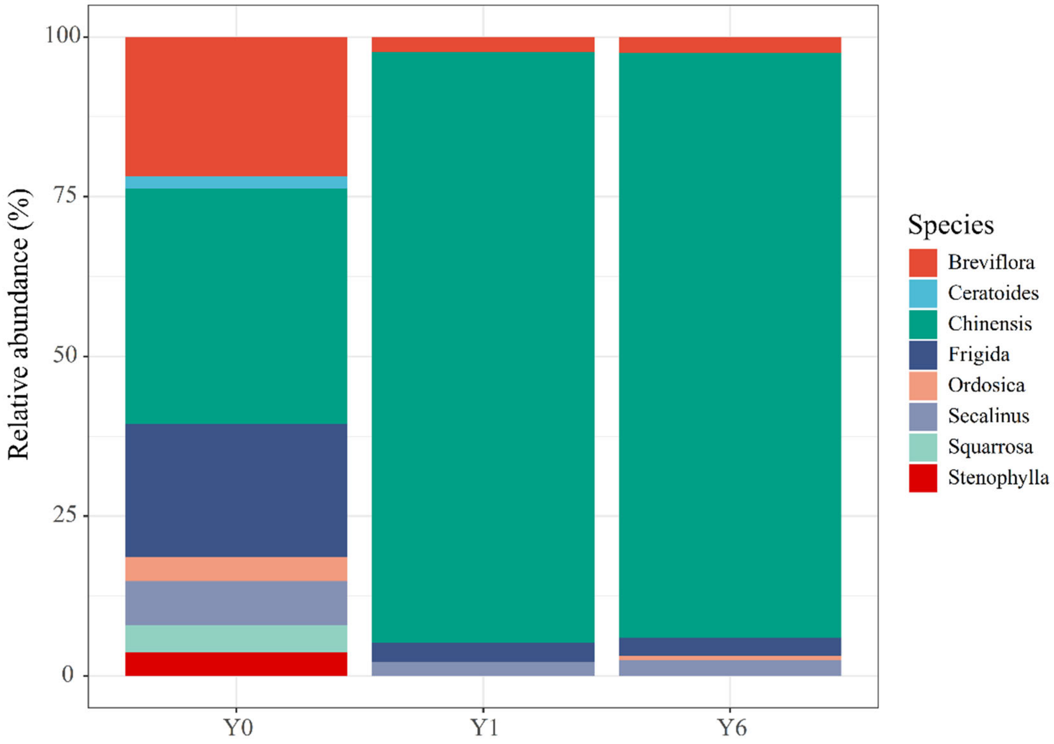

Figure. S13 The differences in community weighted mean of plant height and SLA

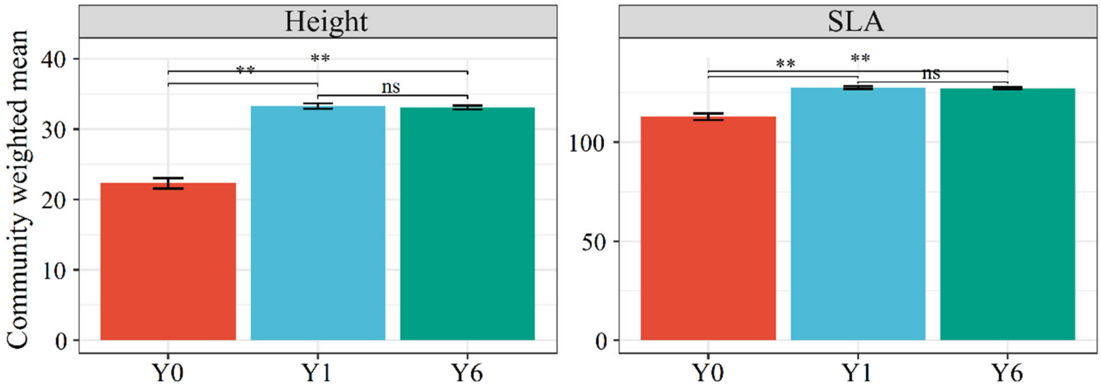

Figure. S14 The standard regression coefficients of plant traits on ecosystem functioning and multifunctionality indices

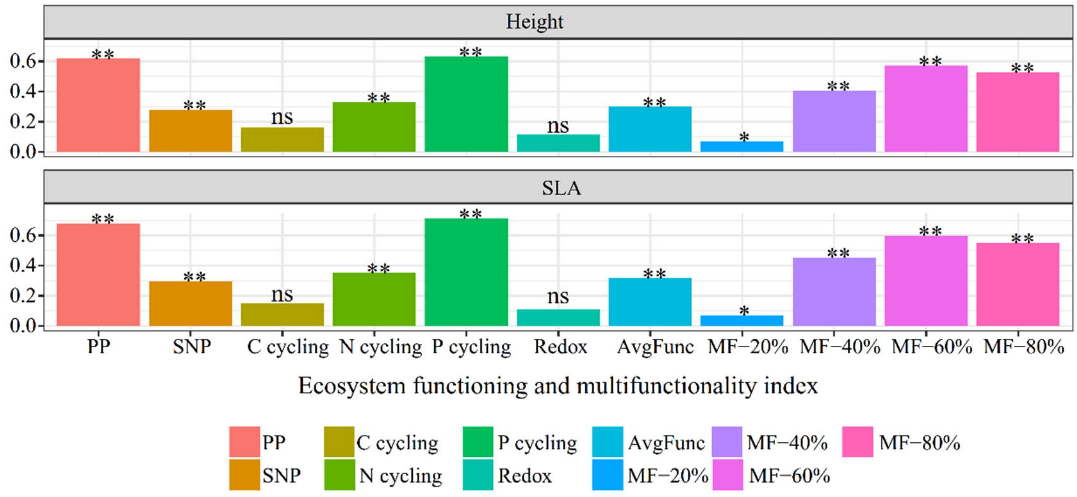

Figure. S15 The correlations among ecosystem functioning and EMF. The cross indicate insignificant correlations at 0.05 level.

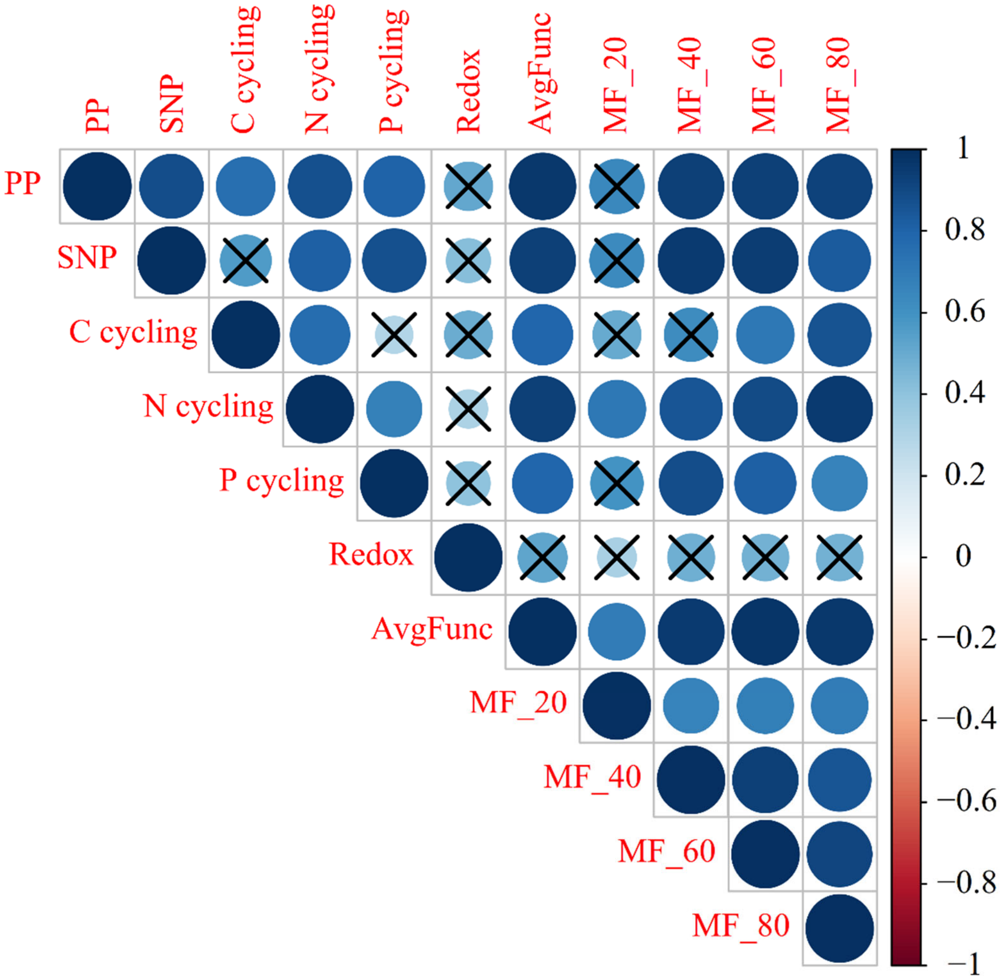

Figure. S16 The correlations between α diversity indices of plant, prokaryote and fungi

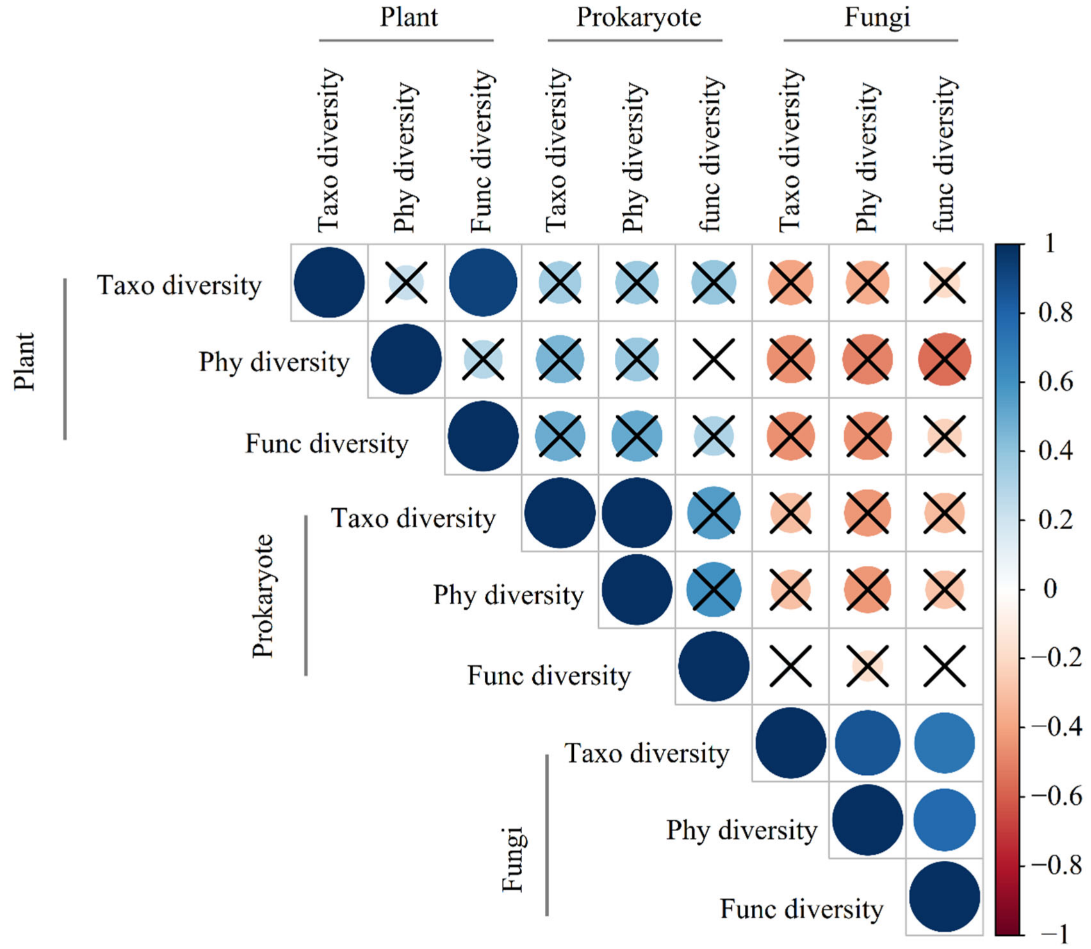

Figure. S17 The correlations between the relative abundance of fungal indicator taxa and soil enzyme activities. * indicates significant correlations at 0.05 level.

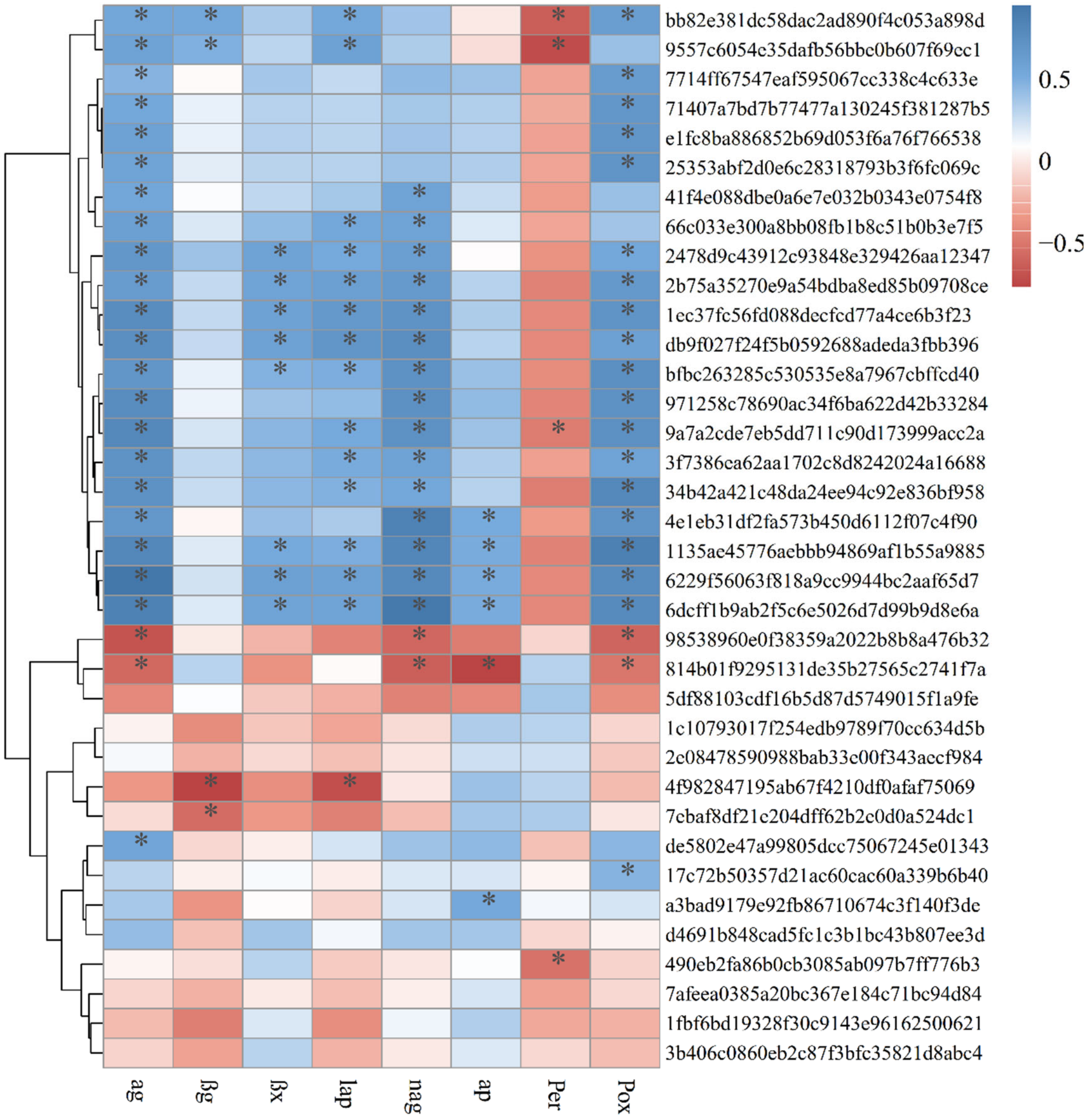

Figure. S18 The changes in the relative abundance of fungal guilds

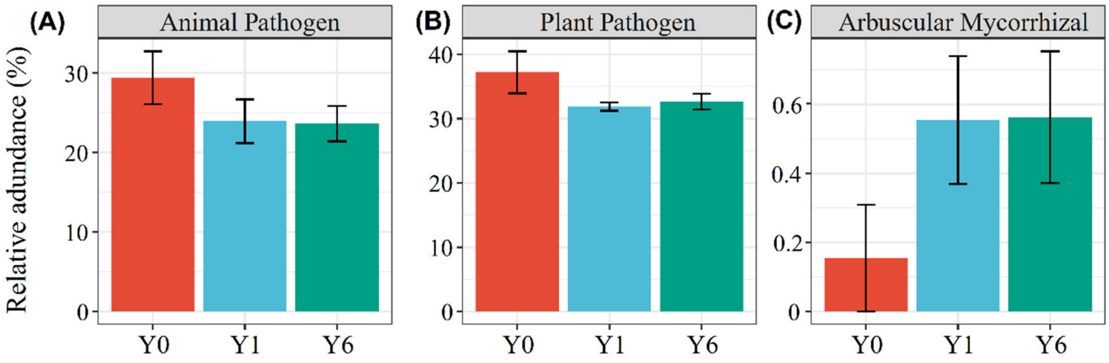

Figure. S19 The correlations between environmental factors and relative abundance of fungal indicator taxa. * indicates significant correlations at 0.05 level.

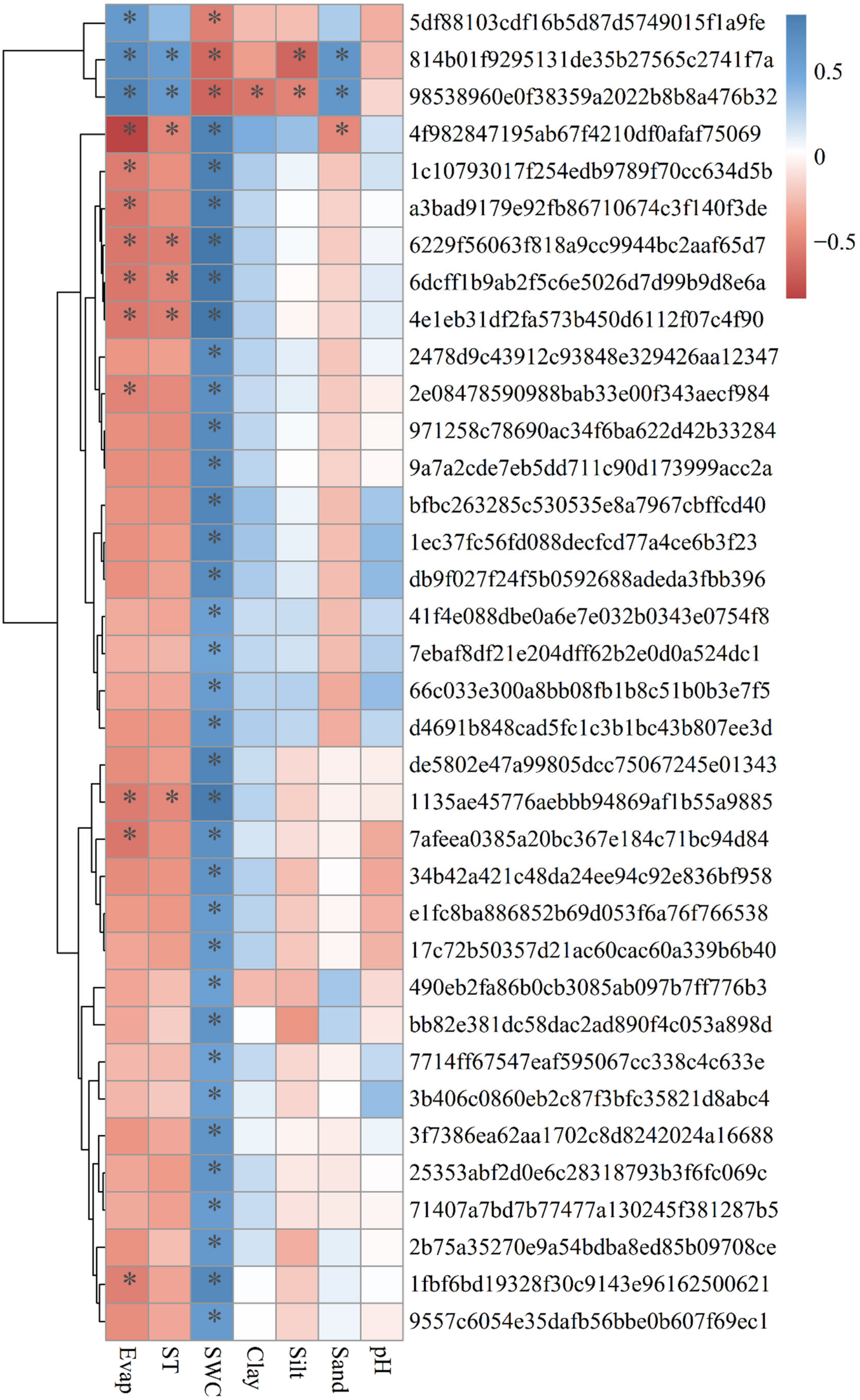
5df88103cdf16b5d87d5749015f1a9fe
814b01f9295131de35b27565c2741f7a
98538960e0f38359a2022b8b8a476b32
4f982847195ab67f4210df0afaf75069
1c10793017f254edb9789f70cc634d5b
a3bad9179e92fb86710674c3f140f3de
6229f56063f818a9cc9944bc2aaf65d7
6dcff1b9ab2f5c6e5026d7d99b9d8e6a
4e1eb31df2fa573b450d6112f07c4f90
2478d9c43912c93848e329426aa12347
2e08478590988bab33e00f343aecf984
971258c78690ac34f6ba622d42b33284
9a7a2cde7eb5dd711c90d173999acc2a
bfbc263285c530535e8a7967cbffcd40
1ec37fc56fd088decfcd77a4ce6b3f23
db9f027f24f5b0592688adeda3fbb396
41f4e088dbe0a6e7e032b0343e0754f8
7ebaf8df21e204dff62b2e0d0a524dc1
66c033e300a8bb08fb1b8c51b0b3e7f5
d4691b848cad5fc1c3b1bc43b807ee3d
de5802e47a99805dcc75067245e01343
1135ae45776aebbb94869af1b55a9885
7afeea0385a20bc367e184c71bc94d84
34b42a421c48da24ee94c92e836bf958
e1fc8ba886852b69d053f6a76f766538
17c72b50357d21ac60cac60a339b6b40
490eb2fa86b0cb3085ab097b7ff776b3
bb82e381dc58dac2ad890f4c053a898d
7714ff67547eaf595067cc338c4c633e
3b406c0860eb2c87f3bfc35821d8abc4
3f7386ea62aa1702c8d8242024a16688
25353abf2d0e6c28318793b3f6fc069c
71407a7bd7b77477a130245f381287b5
2b75a35270e9a54bdba8ed85b09708ce
1fbf6bd19328f30c9143e96162500621
9557c6054e35dafb56bbe0b607f69ec1
0.5
0
−0.5
Evap
ST
SWC
Clay
Silt
Sand
pH

Reference

[1] Bach C E, Warnock D D, Van Horn D J, et al. Measuring phenol oxidase and peroxidase activities with pyrogallol, L-DOPA, and ABTS: effect of assay conditions and soil type[J]. Soil Biology and Biochemistry. 2013, 67: 183-191.
[2] Bridgham S D, Ye R. Organic matter mineralization and decomposition[J]. Methods in biogeochemistry of wetlands. 2013, 10: 385-406.
[3] Delgado-Baquerizo M, Maestre F T, Gallardo A, et al. Decoupling of soil nutrient cycles as a function of aridity in global drylands[J]. Nature. 2013, 502(7473): 672-676.
[4] Dick W A, Tabatabai M A. An alkaline oxidation method for determination of total phosphorus in soils[J]. Soil Science Society of America Journal. 1977, 41(3): 511-514.
[5] Marx M, Wood M, Jarvis S C. A microplate fluorimetric assay for the study of enzyme diversity in soils[J]. Soil biology and biochemistry. 2001, 33(12-13): 1633-1640.
